# Co-segregation of hyperactivity, active coping styles, and cognitive dysfunction in mice selectively bred for low levels of anxiety

**DOI:** 10.3389/fnbeh.2013.00103

**Published:** 2013-08-15

**Authors:** Yi-Chun Yen, Elmira Anderzhanova, Mirjam Bunck, Julia Schuller, Rainer Landgraf, Carsten T. Wotjak

**Affiliations:** Department of Neuronal Plasticity, Max Planck Institute of PsychiatryMunich, Germany

**Keywords:** LAB, selective breeding, hyperactivity, amphetamine, ADHD

## Abstract

We established mouse models of extremes in trait anxiety, which are based on selective breeding for low vs. normal vs. high open-arm exploration on the elevated plus-maze. Genetically selected low anxiety-related behavior (LAB) coincided with hyperactivity in the home cage. Given the fact that several psychiatric disorders such as schizophrenia, mania, and attention deficit hyperactivity disorder (ADHD) share hyperactivity symptom, we systematically examined LAB mice with respect to unique and overlapping endophenotypes of the three diseases. To this end Venn diagrams were used as an instrument for discrimination of possible models. We arranged the endophenotypes in Venn diagrams and translated them into different behavioral tests. LAB mice showed elevated levels of locomotion in the open field (OF) test with deficits in habituation, compared to mice bred for normal (NAB) and high anxiety-related behavior (HAB). Cross-breeding of hypoactive HAB and hyperactive LAB mice resulted in offspring showing a low level of locomotion comparable to HAB mice, indicating that the HAB alleles are dominant over LAB alleles in determining the level of locomotion. In a holeboard test, LAB mice spent less time in hole exploration, as shown in patients with schizophrenia and ADHD; however, LAB mice displayed no impairments in social interaction and prepulse inhibition (PPI), implying a unlikelihood of LAB as an animal model of schizophrenia. Although LAB mice displayed hyperarousal, active coping styles, and cognitive deficits, symptoms shared by mania and ADHD, they failed to reveal the classic manic endophenotypes, such as increased hedonia and object interaction. The neuroleptic haloperidol reduced locomotor activity in all mouse lines. The mood stabilizer lithium and the psychostimulant amphetamine, in contrast, selectively reduced hyperactivity in LAB mice. Based on the behavioral and pharmacological profiles, LAB mice are suggested as a novel rodent model of ADHD-like symptoms.

## Introduction

Animal models appear to be invaluable for understanding the pathophysiology of mental diseases and for hastening the development of therapeutics. The generation of valid animal models of human neuropsychiatric disorders is challenging given the subjective nature of symptoms and the lack of biomarkers and objective diagnostic tests (Nestler and Hyman, 2010). The situation is further complicated by a broad symptom overlap and high comorbidity between diseases. For example, hyperactivity symptoms (i.e., excessive activity, reckless activity, reduced need of sleep, disturbed behavioral inhibition, impulsivity) are common to several neurological and psychiatric disorders, including bipolar disorder (manic state), schizophrenia, and attention deficit hyperactivity disorder (ADHD) (National Institute of Mental Health, 2008; Miro et al., 2012). The above-mentioned disorders also share some other symptoms, such as impaired cognition and increased aggression, as well as several pathogenetic aspects (Hegerl et al., 2010; Miro et al., 2012). These findings suggest that some symptoms may not evolve in isolation, but rather by co-segregation.

There are several ways to deal with symptom overlap in clinical and translational research. To assign individuals to different diagnostic groups, one could check the unique and overlapping endophenotypes of different psychiatric disorders (i.e., face validity). Moreover, previous research in animals and humans has suggested that pharmacological validation is required to determine the type of disease model (i.e., predictive validity). For example, pharmacological treatment with psychostimulants increased general activity in patients with schizophrenia and mania, while exerting a paradoxical “calming effect” in ADHD patients (Greenhill, 1992). Therefore, one could evaluate candidate animal models by screening their responses to different pharmacological agents when the observed phenotypes are not specific to a certain psychiatric disorder.

Since the year 2000, Landgraf and his colleagues started to generate a breeding protocol with CD1 mice that were selected and mated according to their performance on the elevated plus-maze (EPM) to obtain low (LAB), intermediate (NAB), and high (HAB) anxiety-related behavior mice (Landgraf et al., 2007). With selection pressure on low anxiety phenotype, LAB mice are viewed as non-anxious animals, thus being expected to perform at “chance level” in the EPM test (i.e., spending 50% time on open arms). However, against our expectations LAB mice emerged to be novelty-seeking/risk-taking with disturbed behavioral inhibition, as reflected by a preference for visiting the open arms in the EPM test (>60%; Bunck et al., 2009). Selective breeding resulted in differences in innate anxiety, but also in coping styles and locomotion (Ohl et al., 2002; Krömer et al., 2005). For instance, LAB mice show low immobility scores in the tail-suspension test, increased home cage activity (Krömer et al., 2005), and elevated startle responses (Yen et al., 2012) compared to HAB and NAB mice.

**Figure 1:**
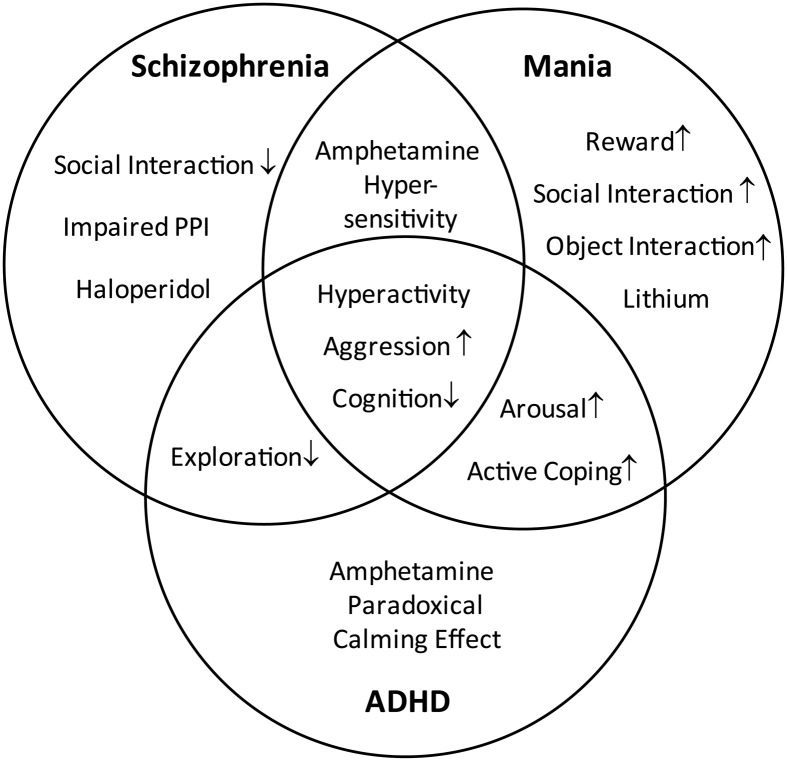
**Distinction and overlap of endophenotypes characteristic for schizophrenia, mania, and ADHD**. The Venn diagram demonstrates the characteristic symptoms of schizophrenia, mania, and attention deficit hyperactivity disorder (ADHD), defined on the basis of DSM-IV. The overlapping areas indicate the common symptoms shared by the disorders.

**Figure 2:**
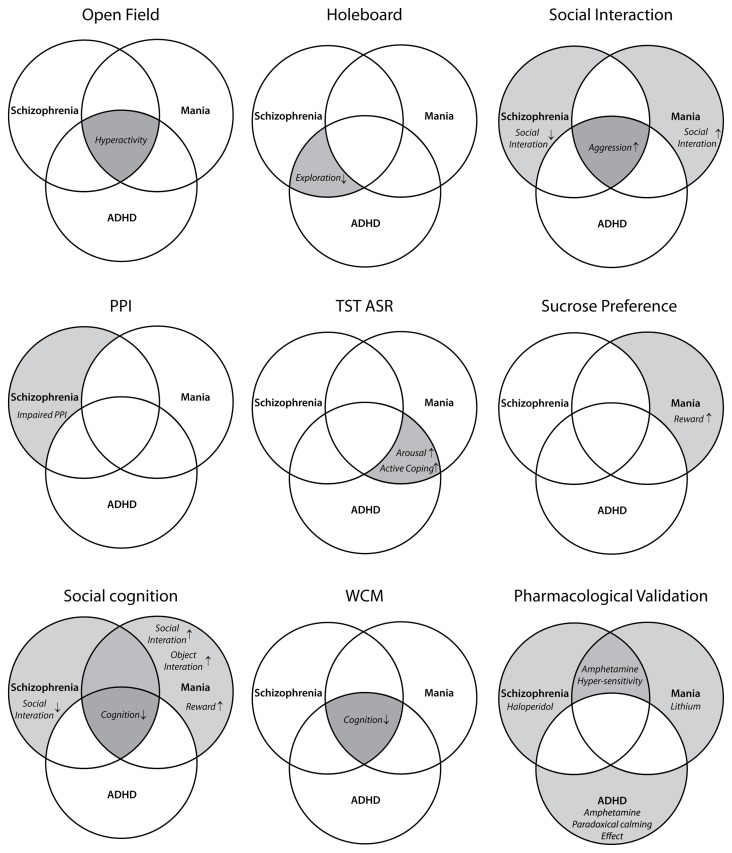
**Assignment of different behavioral tests to the endophenotypic symptoms of schizophrenia, mania, and ADHD**. Each diagram highlights the behavioral traits evaluated by the corresponding test(s) (face validity). OF, open field; HB, holeboard; PPI, prepulse inhibition; TST, tail suspension test; ASR, acoustic startle response; WCM, water cross-maze.

Because of the increased locomotor activity, the present study proposes LAB mice as a model of hyperactivity-related disorders. To validate the type of disease model (i.e., mania vs. schizophrenia vs. ADHD), we first define endophenotypes and pharmacological responses which are shared by the three disorders and/or specific for one of them (Figure 1). Subsequently, we translated them into behavioral tests (Figure 2). Selection of behavioral tests was limited and aimed to illustrate most representative features of endophenotypes. Given the broad spectrum of behavioral and neuroendocrine differences between HAB and LAB mice (Landgraf et al., 2007) and high heritability of general locomotor activity (Logan et al., 2013), F1 hybrids of HAB × LAB vs. LAB × HAB mating were compared in order to figure out the contribution of genetic vs. epigenetic (i.e., maternal behavior) influences on hyperactivity. In addition to the behavioral assessment, responses to the prescribed medications of the three psychiatric disorders were used to further differentiate the psychiatric disorders which commonly possess hyperactive symptoms.

## Materials and methods

### Research strategy

Due to excessive locomotion in home cage and open field (OF), LAB mice are proposed as an animal model of psychiatric disorders which are associated with hyperactivity, such as schizophrenia, bipolar mania, and ADHD. These disorders share a number of symptoms, but differ in certain endophenotypes and pharmacological responses. The similarities and differences are illustrated by a Venn diagram (Figure 1) and translated into different behavioral tests and pharmacological interventions (Figure 2). The subsequent systematical analysis was applied to define the disease model which LAB mice represent.

### Animals

#### HAB/NAB/LAB mice

Male HAB, NAB, and LAB mice used in this study were bred in the animal facilities of the Max Planck Institute of Psychiatry as described previously (Krömer et al., 2005). Briefly, 300 animals of outbred Swiss CD1 mice (Charles River, Sulzfeld, Germany) were used as starting point for selective and bidirectional breeding for extremes in trait anxiety according to their anxiety-related behavior on the EPM. Males and females that spent less than 15% or more than 60% of time on the open arms of the EPM were mated to establish the HAB and LAB mouse lines, respectively. In addition, CD1 mice that spend between 25 and 40% of their time on the open arms were chosen for the selection of NAB mice. All mice of the three lines were tested in the EPM at the age of 7 weeks, with HAB mice spending <15%, NAB mice spending 25–40%, and LAB mice spending >60% of time on the open arms of the EPM. Different batches of inbred HAB, NAB, and LAB males were subjected to various tests in the present study. Data presented were obtained from animals from HAB/LAB generations 29–44 and NAB generations 6–10.

#### F1 hybrids

Generations 40–43 of HAB and LAB mice were used for cross-breeding to generate F1 offspring. HAB females were mated with LAB males (HAB × LAB F1 generation, F1_H × L_, 50 male offspring from 8 litters), and vice versa (LAB × HAB F1 generation, F1_L × H_, 18 male offspring from 8 litters).

#### Housing condition

All mice were single-housed in Makrolon type II cages (23 × 16.5 × 14 cm) ~2 weeks before the experiments started under standard laboratory conditions with reversed 12/12 h light/dark cycle (light on at 9 pm), temperature 23 ± 1°C, food and water *ad libitum*. Laboratory animal care and experiments were conducted according to the regulations of the current version of the German Law and Animal Protection. Animal protocols were approved by the Government of Upper Bavaria (AZ 188-12).

### Elevated plus-maze test

The setup has been described in details (Krömer et al., 2005; Bunck et al., 2009). Briefly, the plus-shaped EPM is made of dark gray PVC and consists of two open (L30 × W5 cm, 300 Lux) and two close arms (L30 × W5 × H15 cm, 10 Lux) connected via a central platform (5 × 5 cm, 90 Lux). The plus-shaped platform was elevated 30 cm above the floor and surrounded by black curtain. At the start of the 5-min trial, the mouse was placed on the central platform facing one of the closed arms. During the 5-min exposure, the percentage of time spent on the open arms and distance traveled were scored using the ANY-maze software (Stoelting Co., USA). In the end of each test, the apparatus was cleaned with detergent-containing water and dried with tissue.

### Open field test

The OF test was performed to measure general exploratory behavior by using the TruScan Photo Beam Activity system (Coulbourn Instruments, Whitehall, PA, USA) as described previously (Jacob et al., 2009). Mice were put onto the center of a Plexiglas cage (L26 × W26 × H38 cm, 10 Lux) for 80-min testing. Each test cage, including the sensor rings, was surrounded by walls made of opaque Plexiglas (L47 × W47 × H38.5 cm). Horizontal and vertical locomotion were automatically recorded by 2 photobeam sensor rings (2 and 5 cm above the floor; photobeams are spaced apart by 1.52 cm providing a 0.73 cm spatial resolution). The distance traveled, rearing activity and mobility time were recorded and analyzed (sampling rate 4 Hz) by TruScan Software Version 1.1 (Coulbourn Instruments). Animals were removed from the test cages in the end of the test and Plexiglas cages were cleaned with detergent-containing water.

In case of pharmacological treatment, the OF test was automatically interrupted for a drug injection procedure and continued by pressing the “start” bar right after the injection.

### Hole-board test

The hole-board (HB) test was also preformed using the TruScan Photo Beam Activity system as described before. For the HB test, a nose poke floor was inserted into the OF Plexiglas cage. Nose-poke behavior can be detected by an additional photobeam sensor ring. Each nose poke floor contains 16 holes (4 × 4 arrays) with 16 corresponding underlying food trays (not filled in the present study). At the beginning of the HB test, animals were placed onto the center of the nose poke floor (15 Lux). Because TruScan software records coordinates by sensor rings, it is possible to analyze the number of holes accessed as well as the sequence in which the holes were accessed. The accuracy of performance was defined as the percent numbers of 16 holes which had been visited at least one time over the course of the exposure, and it was calculated as [(number of holes visited at least once/16) × 100%]. The number of nose-poke entries and accuracy were used to test exploratory behavior in the “immediate” testing environment of the animals.

### Social interaction test

This test was performed as previously described (Smit-Rigter et al., 2010; Terzian et al., 2011). A male BALB/c mouse was put into the home cage (27 × 16 × 12 cm) of the subject mouse at illumination of 5 lux (red light). The wire lid residing food and water bottle and the curly sawdust were removed to avoid the animals hiding out of sight while conducting the test. The two animals were allowed to interact in subject's home cage for 5 min. The time spent in active social interactions (sniffing, licking, close following, and grooming) and social aggression/attack (biting and fighting) of the subject mouse were recorded and scored off-line by using the computer software Eventlog 1.0 (designed by Robert Henderson, 1986).

### Prepulse inhibition test

Prepulse inhibition (PPI) refers to the attenuation of the startle reflex by a weak prepulse, which precedes the startling stimulus by 25–100 ms. Mice were placed into one out of eight identical startle apparatus which consists of a non-restrictive Plexiglas cylinder (inner diameter 4 cm, length 8 cm) mounted onto a plastic platform. Each startle apparatus was housed in a sound attenuated chamber (SR-LAB, San Diego Instruments SDI, San Diego, CA, USA) with a continuous 50 dB background noise. Startle pulses and background noise were delivered through a speaker 20 cm above each Plexiglas cylinder. The movements of cylinder were detected by a piezoelectric element. The voltage output of the “piezo” was amplified and then digitized (sampling rate 1 kHz) by a computer interface (I/O-board provided by SDI). Startle amplitude was taken as the highest voltage during a time window of 20 ms. Mice were acclimated to the startle apparatus for 5 min before the first trial began. The first 20 trials consist of 20 startle pulses (white noise 115 dB) which served to habituate and stabilize the animals' startle response and which were not included in the analysis. Each session consisted of the following: 22 pulse-alone trials (115 dB), 210 prepulse (PP)-condition trials, and 18 prepulse-alone trials. The 250 discrete trials were presented in a pseudorandom order, with a variable inter-trial interval of a 15 s on average (ranging from 13 to 17 s). Fifteen different prepulse-condition trials were presented, each for 14 times. Three different prepulse intensities were adopted (55, 65, or 75 dB white noise; background noise: 50 dB) with an inter-pulse interval (IPI, between onsets of the prepulse and pulse) of 5, 10, 25, 50, or 100 ms. The duration of the prepulse was 10 or 5 ms when the IPI was 5 ms. PPI was calculated as follows: % PPI = [(PP-condition – pulse-alone)/pulse-alone × 100%].

### Acoustic startle response

To test for arousal levels, acoustic startle responses (ASR) were measured automatically as described before (Golub et al., 2009; Yen et al., 2012). The session began with placing the animals into a Plexiglas enclosure. Mice were acclimated to the apparatus for 5 min and then presented with 136 startle pulses with an IPI of 13–17 s. The intensities of white noise were 75, 90, 105, and 115 dB with 30 startle trials at each level in a pseudorandom order. Additionally, animals' startle responses were recorded for 16 times under background noise. At the end of each test, Plexiglas cylinders were cleaned thoroughly with soap water.

### Tail suspension test

The details of tail suspension test (TST) are described previously (Krömer et al., 2005; Bunck et al., 2009). Each tested animal was suspended by the tip of tail with an adhesive tape to a rod that was 35 cm above the ground for 6 min. Four animals were tested at the same time. The animals were not separated by partitions during the test and each trial contained only one phenotype. Each trial was videotaped and the immobility time was analyzed by an observer blind to the mouse line or treatment using the computer software Eventlog 1.0 (designed by Robert Henderson, 1986).

### Sucrose consumption test

Mice had a free access to two bottles, one with sucrose solution (2.5%) and another with tap water, for 10 h (during the dark phase) per day for two consecutive days (Strekalova et al., 2005; Terzian et al., 2011). The animals were not food or water-deprived before the test. One day prior to the test day, animals were habituated to drink sucrose solution (2.5%) for 2 h. To prevent possible effects of side preference in drinking behavior, the bottle positions were swapped in the mid-point of testing (after 5 h). The consumptions of water and sucrose solution were estimated by weighing the bottles before and after each trial. To compare sucrose preference between the different mouse lines, we additionally expressed sucrose consumption as a percentage of the total fluid intake.

### Social cognition

#### Social preference test

The social preference test was conducted in a rectangular box made of white PVC walls and a darkgray PVC floor. The box was divided in three compartments (L30 × W30 × H30 cm each) which were connected by two opening doors (6 × 5 cm). The social approach behaviors were performed using essentially the same procedures as previously described (Moy et al., 2004; Nadler et al., 2004; Crawley et al., 2007) with minor modifications. In brief, the test consisted of four 10-min trial sessions (Figure 10A). During the first 10 min (figure not shown), mouse was placed into the center compartment with both doors closed, in order to familiarize the subject mouse with the testing environment and the center compartment. During the next 10 min (Figure 10A1), the doors were open and the subject mouse could habituate to three compartments and two empty perforated 50 ml plastic tubes (SARSTEDT AG & Co., Nürmbrecht, Germany) each placed in the center of a side compartment. The third 10 min served as a sampling session (Figure 10A2), during which one empty tube was replaced by an identical tube which contained an ovarectomized female (FM1) mouse. This 10 min session was designed to see difference between the sniffing time spent in social stimulus and non-social stimulus. The last 10 min period was the testing session (Figure 10A3), during which another empty tube was replaced by an identical one containing a novel ovarectomized female (FM2). This session was designed to test the ability of the subject mouse to distinguish two female individuals.

#### Social discrimination test

The social discrimination established in rats (Engelmann et al., 1995) was adopted for mice (Richter et al., 2005). Figure 10B1 depicts the experimental procedures. The hypothesis behind this test is similar to social preference test. Compared to social preference test, this is task is more challenging for the experimental animals because they have to distinguish two stimulus females after a certain period of time. After being transferred to the experimental cage (L25 × W22 × H38 cm) with an empty perforated 50 ml plastic tube for 60 min of habituation, the subject animal was introduced to the first stimulus female (FM1), protected in a perforated plastic tube for 5 min. After an inter-exposure interval (IEI) of 15 min, 30 min, 2h, or 4 h respectively, the first (FM1) ovarectomized female was reintroduced for 5 min to the test mouse together with a novel (FM2) stimulus animal (also in a plastic tube). A significant increase in olfactory investigation of the novel female compared to the familiar female during the second exposure was taken as a measure of the animals' social discrimination ability/social memory (Engelmann et al., 1995).

Both social cognitive experiments were videotaped for later analysis. The time spent in social interactions, aggressive behavior or olfactory investigation towards the respective stimulus animal was quantified off-line by using the computer software Eventlog 1.0 (designed by Robert Henderson, 1986). In the end of test, setups were cleaned thoroughly with soap water.

### Water cross-maze test

The water cross-maze (WCM, custom made, Max Planck Institute of Psychiatry, Germany) was performed essentially as described (Kleinknecht et al., 2012). The WCM is made of 1-cm thick transparent Plexiglas and consists of four identical arms (L50 × W10 × H30 cm; corresponding to North, East, South, and West arms in clockwise order). A Plexiglas platform (L9 × W9 × H10 cm) was located at the end of West or East arms depending on the testing phases. The maze was filled with fresh tap water at 23°C before testing. The top of the platform was submerged 1.5 cm below the water surface, so that it was invisible to the animals. The testing room was illuminated by indirect spectrum light of four lamps, resulting in light intensity of 20 Lux at the upper edge of the maze and 14 Lux at the level of water surface. The room contained sufficient distal visual cues (i.e., sink, small gray cabinet, etc.) for animals to orient during the test. In each testing trial, after placing animals into the maze, the experimenter consistently stood at the same position (the end of South arm) to avoid any alteration in the testing environment. Mice were kept in a holding room adjacent to the testing room and transported to the testing room for each trial.

The animals were trained in groups of six for six trials per day with equal inter-trial intervals of 10 min in the free learning (FL) protocol (Kleinknecht et al., 2012). The FL protocol is a dual-choice protocol, which allows animals to locate platform position by using either place-based allocentric or response-based egocentric strategy. In the first week of 4- or 5-day training, the North arm was always blocked by a partition during testing and animals were trained to swim from the end of the South arm and navigate the platform at the end of the West arm. In the second week during reversal training, the platform was transferred to the East arm, and the animals started from the same position (South arm), but need to shift their navigation from the West to the East arm. In each trial, mice were given 30 s to find the submerged platform. The time from placing the animal into the water until it has reached on the platform was measured as escape latency. If the mouse failed to reach the platform by 30 s, it was guided onto the platform by a metal stick, and a score of 31 s was assigned for that trial. After mounting on the platform, the animals were allowed to remain there for 5 s, and were then withdrawn by the metal stick back to their home cages until the start of next trial.

In addition to the escape latency, accuracy and wrong platform visits were taken as measures of animals' learning performance in the WCM. The trial was recorded as accurate if the animals mounted on the platform in the goal arm without visiting the arm opposite to the goal arm or reentering the start arm within 30 s. Accuracy was defined as the percent accurate trials out of 6 testing trials per day [(sum of correct trials/6) × 100%]. Animals were assigned as accurate learners if they performed accurately in 5 or 6 out of 6 trials per day (Kleinknecht et al., 2012). Wrong platform visits were counted when the animal entered the outer third of the arm opposite to the goal arm.

### Drugs

For systemic drug administration, mice were treated intraperitoneally (i.p.) with 1.0 mg/kg haloperidol (Halol, Jassen-Cilag GmbH, Neuss, Germany), 100 mg/kg lithium chloride (Sigma-Aldrich, USA), 1.0 or 2.0 mg/kg d-amphetamine sulfate (Sigma-Aldrich, USA) dissolved in sterile saline with a volume of 0.1 ml/10 g.

### Data analysis and statistics

Data are shown as mean ± S.E.M. Statistical analyses were performed using Statistica 5.0 (StatSoft, Inc., Tulsa, OK, USA).

Statistical evaluation of OF, HB, social interaction, PPI, ASR, TST, sucrose consumption, and WCM tests was performed by either One-Way or Two-Way ANOVA with repeated measures followed by a *post-hoc* Newman–Keuls test. Social cognitive functions were analyzed by dependent *t*-test separately per line. For the matter of clarity, in a few experiments, we refrained from showing the results of *post-hoc* analyses in the figures. Instead, we mentioned them in the text. A *p* < 0.05 was accepted as statistically significant.

## Results

### LAB mice show elevated levels of locomotion without habituation

#### Mice bred for low anxiety-related behavior (LAB) display increased locomotor activity

**Figure 3:**
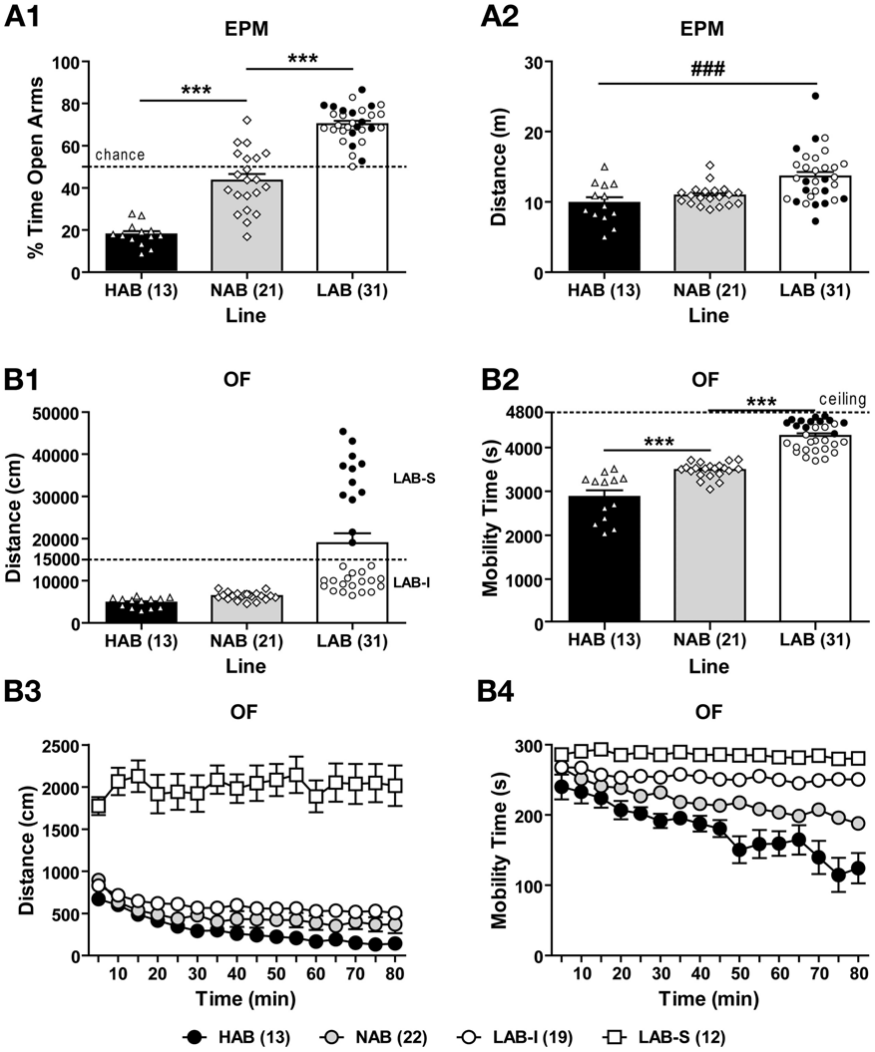
**Hyperlocomotion in low anxiety-related behavior (LAB) mice. (A1)** Mice originating from selectively bred lines showed low (LAB), intermediate (NAB), or high (HAB) levels of innate anxiety on the elevated plus-maze (EPM). **(A2)** LAB mice were more active than HAB mice in the EPM test. **(B1)** In the open field (OF) test (80-min exposure), LAB mice traveled longer distance compared to HAB and NAB mice. In addition, they showed two clusters of total distance traveled which could be distinguished by a threshold of 15000 cm (dashed line), revealing LAB-intermediate (LAB-I; open circles) and LAB-strong (LAB-S; close circles) mice. Interestingly, LAB-I and LAB-S mice did not differ in anxiety-related behavior and distance traveled assessed in the EPM test (cf. **A1** and **A2**). **(B2)** LAB-I mice spent significantly more time in mobility than HAB and NAB mice, but less than LAB-S mice. Exploratory behavior over the course of testing was measured in terms of **(B3)** distance traveled and **(B4)** mobility time in 5-min bins for the 80-min exposure. LAB-S mice displayed much higher levels of distance traveled than HAB, NAB, and LAB-I mice, with an additional significant difference between LAB-I and HAB mice. In addition to distance traveled, mobility time also significantly differed between all four lines. Notably, in contrast to pronounced habituation to the OF in HAB, NAB, and LAB-I mice, LAB-S mice showed virtually no habituation over the course of the entire exposure, if locomotion and mobility were considered. Mean ± SEM. ^***^p < 0.001 vs. NAB; ^###^*p* < 0.01 vs. HAB (ANOVA followed by *post-hoc* Newman–Keuls test).

HAB (*n* = 13), NAB (*n* = 21), and LAB (*n* = 31) mice were tested in the EPM and OF tests. The difference in innate anxiety between HAB, NAB, and LAB mice was confirmed in the EPM [*F*_(2, 62)_ = 127.04, *p* < 0.001]. *Post-hoc* comparisons revealed that HAB mice spent lower and LAB mice higher percent time on the open arms than did NAB animals (*p* < 0.05; Figure 3A1). We also measured the distance traveled as an index of EPM-related locomotion, which indicated significant differences between lines [*F*_(2, 62)_ = 5.26, *p* < 0.01]. *Post-hoc* analyses revealed higher levels of distance traveled in LAB mice compared to HAB mice (*p* < 0.05; Figure 3A2).

In the OF during the entire 80-min observation period, HAB, NAB, and LAB mice displayed differences in the total distance traveled [*F*_(2, 62)_ = 17.32, *p* < 0.001; Figure 3B1] and total mobility time [*F*_(2, 62)_ = 86.64, *p* < 0.001; Figure 3B2]. Analysis of the individual data revealed that in the LAB population, two clusters of data points were observed which traveled either more (LAB-strong, LAB-S) or less (LAB-intermediate, LAB-I) than 15,000 cm (Figure 3B1). Notably, further analysis regarding the LAB subgroups revealed that LAB-S and LAB-I mice differed in mobility time in the OF as well [unpaired *t*-test: *t*_(29)_ = 6.72, *p* < 0.01; Figure 3B2], but not in anxiety and locomotion in the EPM (cf. distribution of LAB-I and LAB-S mice in Figures 3A1,A2). Analysis of distance traveled over the course of OF exposure revealed significant differences between lines [*F*_(3, 61)_ = 116.44, *p* < 0.001] with a significant line × time interaction [*F*_(45, 915)_ = 3.54, *p* < 0.001; Figure 3B3]. *Post-hoc* comparisons indicated that LAB-S mice differed significantly from the other three lines (*p* < 0.001). LAB-I mice displayed significantly more distance traveled compared to HAB mice (*p* < 0.05), but only a tendency for increased locomotion compared to NAB mice (*p* = 0.058). The One-Way ANOVA revealed that HAB, NAB, and LAB-I displayed pronounced short-term habituation [*F*_(15, 180)_ = 46.90, *p* < 0.001 for HAB; *F*_(15, 300)_ = 107.87, *p* < 0.001 for NAB; *F*_(15, 255)_ = 8.65, *p* < 0.001 for LAB-I], whereas LAB-S mice showed no habituation over the course of OF exposure (*F* < 1). The mobility time over the course of OF exposure (Figure 3B4) significantly differed between all four lines [*F*_(3, 61)_ = 89.14, *p* < 0.001] with a significant line × time interaction [*F*_(45, 915)_ = 5.18, *p* < 0.001]. LAB-S mice spent significantly more time being mobile compared to the other three lines (all *p* < 0.001), as well as LAB-I exhibited more mobility time than both NAB and HAB mice (both *p* < 0.001). Moreover, significant difference was also found between NAB and HAB mice (*p* < 0.001). Likewise, the One-Way ANOVA in mobility revealed that HAB, NAB, and LAB-I displayed short-term habituation [*F*_(15, 180)_ = 6.60, *p* < 0.001 for HAB; *F*_(15, 300)_ = 25.42, *p* < 0.001 for NAB; *F*_(15, 255)_ = 2.90, *p* < 0.001 for LAB-I], whereas LAB-S mice showed no habituation over the course of OF exposure [*F*_(15, 180)_ = 1.06, *p* > 0.05].

#### LAB mice display persistence in hyperactivity without habituation upon repeated testing

**Figure 4:**
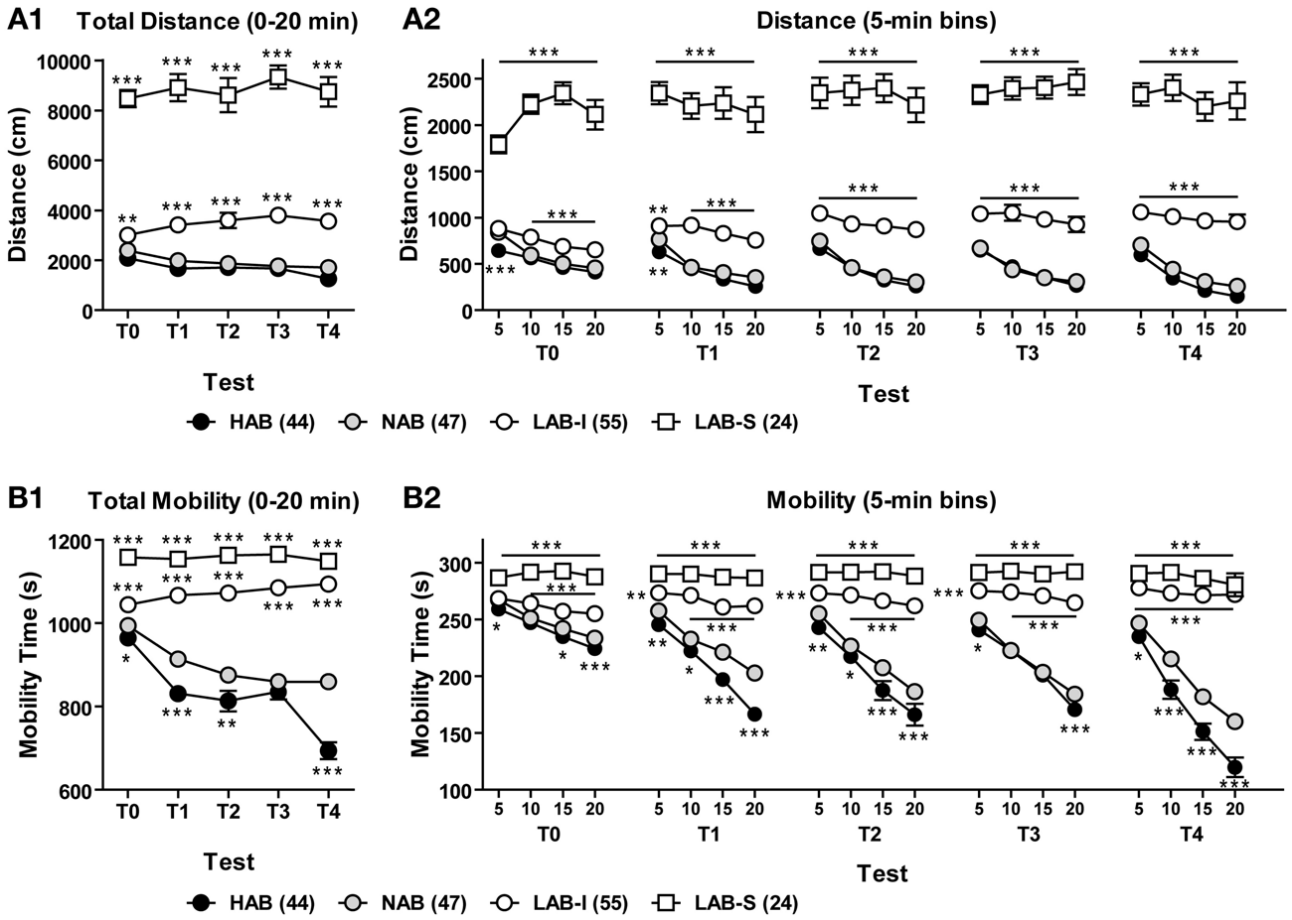
**LAB mice lack habituation upon repeated OF testing**. Animals were repeatedly tested in the OF test for 20 min in 5 sequential tests (T0–4). **(A1,B1)** Over the course of repeated testing, HAB and NAB mice showed a pronounced decrease between tests (long-term habituation) in total distance traveled and mobility time, while LAB-I and LAB-S failed to do so. Notably, LAB-I mice even traveled farther and moved more across repeated testing. **(A2,B2)** HAB and NAB mice habituated to the OF within the first 20 min (short-term habituation) of each test and displayed more reduction in distance traveled and mobility time after repeated testing. LAB-I mice showed short-term habituation during the first test (T0), but failed to display short-term habituation after repeated testing. LAB-S revealed no short-term habituation at all as reflected by persistent high levels of distance traveled and mobility time. Mean ± SEM. ^*^p < 0.05, ^**^p < 0.01, ^***^p < 0.001 compared to NAB mice; “line and asterisk” refer to all symbols above/below (Two-Way ANOVA followed by *post-hoc* Newman–Keuls test).

To assess the persistence of line differences in exploratory behavior, we reanalyzed the OF data from several batches of animals that were repeatedly tested for 20 min in 5 sequential tests (T0–4). Total distance traveled and total mobility time during the 20-min OF exposure of each test were analyzed to evaluate long-term habituation (Figures 4A1,B1). The Two-Way ANOVA with repeated measures revealed a main effect of line in distance traveled [*F*_(3, 102)_ = 272.41, *p* < 0.001] with a significant line × test interaction [*F*_(12, 408)_ = 3.44, *p* < 0.001]. Further analyses indicated that LAB-S mice differed significantly from the other three lines (all *p* < 0.001) and LAB-I displayed more distance traveled in comparison with HAB and NAB mice in all 5 tests (both *p* < 0.001). The difference between LAB-I and HAB/NAB lines was more pronounced after repeated testing. Analyses of mobility time revealed essentially the same as the findings of distance traveled (Figure 4B1; statistics not shown).

Figures 4A2,B2 illustrate the distance traveled and mobility time measured in 5-min bins during the 20-min exposure in the 5 sequential OF tests. For both distance traveled and mobility, the Two-Way ANOVAs revealed main effects of line (*p* < 0.001) and trial (*p* < 0.001) as well as significant line × trial interactions (*p* < 0.001) in all 5 tests. *Post-hoc* comparisons showed significant differences between HAB/NAB, LAB-I, and LAB-S mice (all *p* < 0.001), but no difference between HAB and NAB mice. Further, the One-Way repeated measures ANOVA indicated habituation in HAB and NAB mice, which was not the case in LAB-S and LAB-I mice (upon repeated testing).

#### LAB-S mice are equally distributed among the different lab breeding pairs

**Figure 5:**
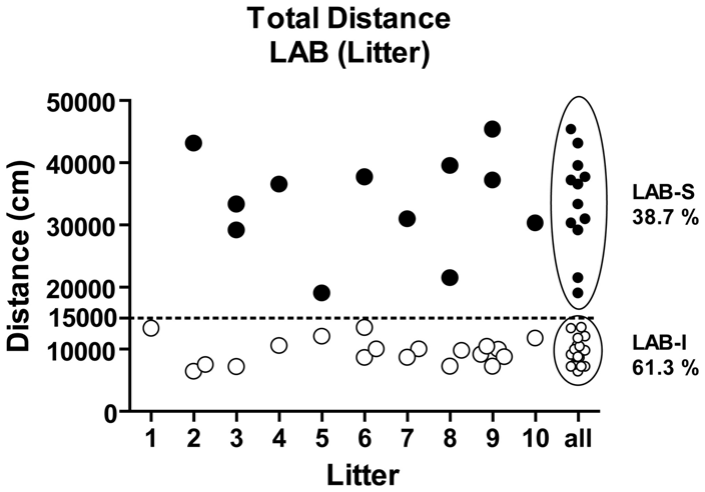
**LAB mice by different litters**. Analysis of LAB's individual total distance traveled by litter revealed that LAB-S mice were evenly distributed across different breeding pairs.

Given the fact that the LAB population splits up into two subgroups (Figure 5), we were curious whether LAB-S mice originated from particular breeding pairs. Figure 5 shows the total distance traveled by different litters (random selection). In each litter, the percentage of LAB-S offspring ranged form 0 to 67.7% with no evidence for accumulation of LAB-S mice in particular litters. Based on the cut-off criterion (15,000 cm), 13 out of 31 LAB mice (38.7%) were assigned as LAB-S and 18 out of 31 (61.3%) as LAB-I mice.

#### F1 generations do not show lab-like phenotype in locomotion

**Figure 6:**
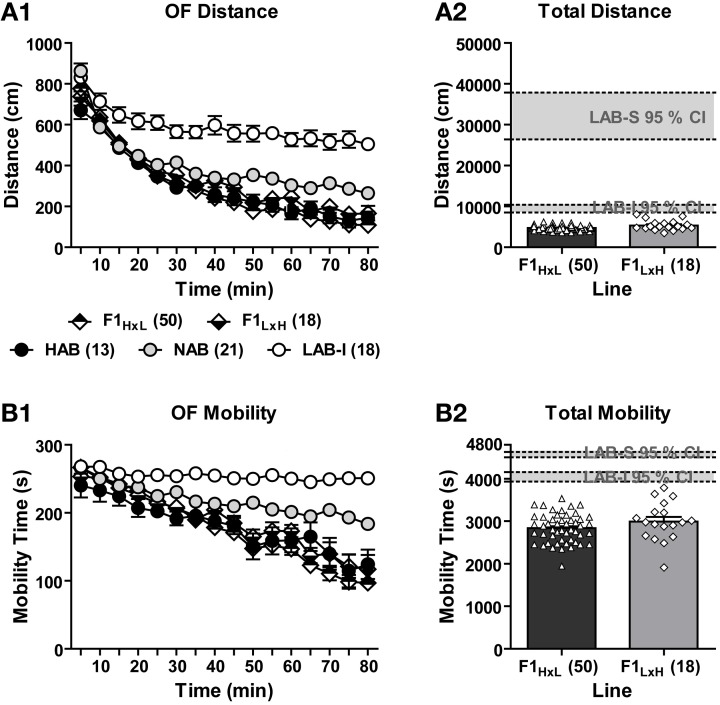
**Locomotor activity in F1_H × L_ vs. F1_L × H_ mice**. In an 80-min OF test, F1 hybrids originating from HAB × LAB (F1_H × L_) and LAB × HAB (F1_L × H_) breeding exhibited the same level of **(A1)** distance traveled and **(B1)** mobility time that was much lower than the level of NAB and LAB-I mice. Concerning the **(A2)** total distance traveled and **(B2)** total mobility time, no F1 hybrid fell in the 95% confidence intervals (CI; gray block) of LAB-I- or LAB-S-like phenotypes in locomotion and mobility.

To estimate the contribution of maternal vs. paternal alleles to the line-specific differences in exploratory behavior and the genetic penetrance of the LAB phenotypes, HAB females were mated with LAB males, and vice versa to create two respective F1_H × L_ and F1_L × H_ hybrids. Both hybrid offspring shared 50% of HAB and LAB genetic background. The two F1 hybrid lines were tested in the OF and their exploratory behavior was measured and compared to that of LAB-I, NAB, and HAB mice. During the entire 80-min OF exposure, the five lines significantly differed in the distance traveled [*F*_(4, 115)_ = 64.20, *p* < 0.001; Figure 6A1] and mobility time [*F*_(4, 115)_ = 50.27, *p* < 0.001; Figure 6B1]. *Post-hoc* comparisons revealed that no significant difference was found between F1_H × L_, F1_L × H_ and HAB mice, whereas NAB and LAB-I mice showed significantly more distance traveled and mobility time compared to F1_H × L_ and F1_L × H_ mice (*p* < 0.01). Further analyses of total distance traveled and mobility time revealed that none of F1 mice fell in the 95% confidence intervals of LAB-I and LAB-S mice (Figures 6A2,B2). Collectively, these data speak for the dominance of the HAB genetic background over LAB and against a preeminence of maternal behavior in shaping the LAB phenotypes, at least on a F1 hybrid background.

### LAB mice spent less time exploring the immediate testing environment

**Figure 7:**
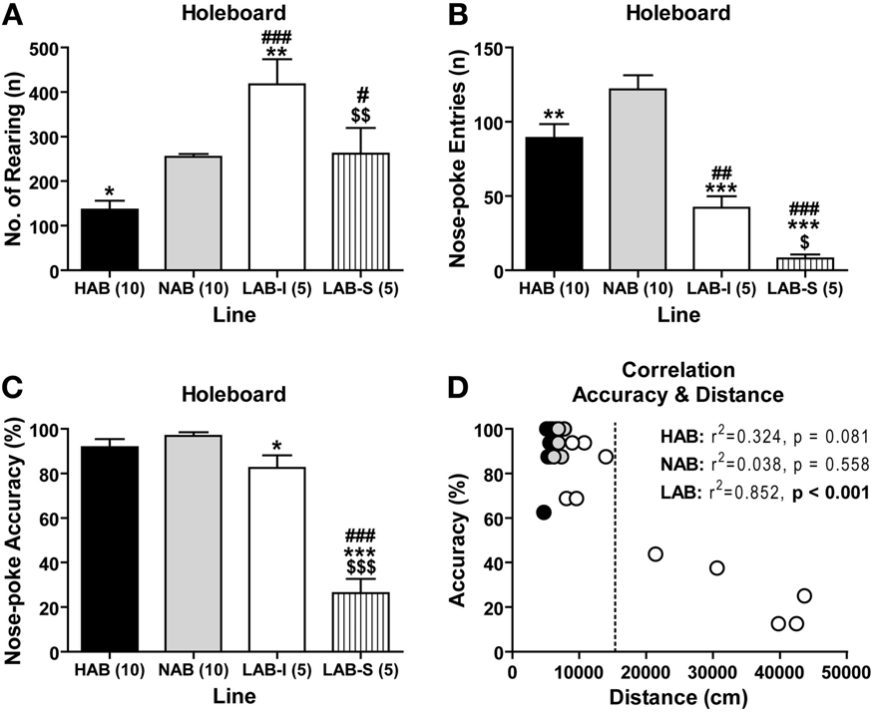
**LAB mice displayed a reduction in hole exploration**. In the hole-board test (30-min exposure), **(B)** LAB-I and LAB-S mice showed less downward hole exploration than HAB and NAB, which was different from the **(A)** upward vertical exploration (i.e., number of rearing). **(C)** Compared to HAB, NAB, and LAB-I mice, LAB-S mice showed the poorest performance in nose-poke accuracy, which describes how many of the 16 holes had been visited at least once over the course of the exposure. **(D)** Intriguingly, there was an inverse relationship between locomotor activity and accuracy of hole exploration in LAB mice, but not in HAB and NAB mice. Note that LAB-I and LAB-S could also be distinguished in the hole-board by the threshold of 15,000 cm (dashed line). Mean ± SEM. ^***^p < 0.001; ^**^p < 0.01; ^*^p < 0.05 *vs*. NAB. ^###^*p* < 0.001; ^#^*p* < 0.05 *vs*. HAB. ^$$$^*p* < 0.001; ^$$^*p* < 0.01; ^$^*p* < 0.05 *vs*. LAB-I (ANOVA followed by *post-hoc* Newman–Keuls test).

To evaluate how the animals respond to a more complex environment, we tested 10 HAB, 10 NAB, 5 LAB-I, and 5 LAB-S mice in a modified OF, which contained a floor with 16 holes (HB test). Experiments were performed under low-light conditions. The resulting low emotional load was expected to minimize the influence of anxiety on exploratory behavior. Analysis of upward vertical exploration (the number of rearing) revealed significant line differences [*F*_(3, 26)_ = 13.66, *p* < 0.001; Figure 7A]. *Post-hoc* comparisons confirmed that LAB-I mice showed more rearing (exploration of more “distant” testing environment) compared to the other three lines (*p* < 0.01). In contrast, LAB-I and, in particular, LAB-S mice showed decreased downward 16-hole exploration (exploration of more “immediate” testing environment) compared to HAB and NAB mice. For the frequency of nose poking (nose-poke entries), significant differences were found among lines [*F*_(3, 26)_ = 25.11, *p* < 0.001; Figure 7B], and *post-hoc* analyses revealed that LAB-I and LAB-S mice displayed significantly less hole exploration compared to HAB and NAB mice (*p* < 0.01). Not only the frequency of nose poking was reduced in LAB-I and LAB-S mice, but also the accuracy of performance, which describes how many of the 16 holes had been visited at least once over the course of the exposure [*F*_(3, 26)_ = 55.92, *p* < 0.001, Figure 7C]. *Post-hoc* analyses revealed that LAB-S mice showed the poorest performance in nose-poke accuracy compared to the other three lines. Moreover, the nose-poke accuracy was lower in LAB-I than in NAB mice (*p* < 0.05). There was an inverse relationship between locomotor activity and accuracy of hole exploration in LAB (*r*^2^ = 0.852, *p* < 0.001; Figure 7D), but not in HAB and NAB mice.

### LAB mice show no deficits in social interaction and PPI

**Figure 8:**
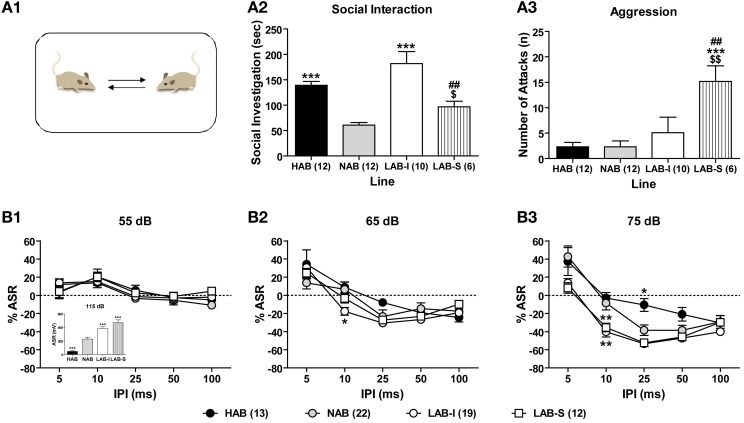
**LAB mice showed no deficits in social interaction and PPI. (A1)** In a social interaction test, **(A2)** LAB-I mice spent more time in social investigation than the other three lines; however, **(A3)** LAB-S mice displayed more aggression compared to the other lines. Mean ± SEM. ^***^p < 0.001 *vs*. NAB. ^##^*p* < 0.01 *vs*. HAB. ^$$^*p* < 0.01; ^$^*p* < 0.05 *vs*. LAB-I (ANOVA followed by *post-hoc* Newman–Keuls test). Mice of four lines were tested in a PPI test. LAB-I and LAB-S mice displayed higher, and HAB mice lower startle response to startle pulses of 115 dB (inset). Change of startle response (% ASR) at PP intensities of **(B1)** 55 and **(B2)** 65 dB did not differ between four lines. **(B3)** At PP intensity of 75 dB, both LAB-I and LAB-S mice showed pronounced PPI with IPI of 10 ms, while HAB mice revealed less PPI with IPI of 25 ms, compared to NAB mice. Mean ± SEM. ^*^p < 0.05; ^**^p < 0.01 *vs*. NAB (ANOVA followed by *post-hoc* Newman–Keuls test).

Social interaction test was conducted to measure levels of social interests and social aggression by confronting the animals with a male BALB/c mouse in their home cages (Figure 8A1). During the 5-min social interaction test, HAB (*n* = 12), NAB (*n* = 12), LAB-I (*n* = 10), and LAB-S (*n* = 6) differed significantly in social exploration [*F*_(3, 36)_ = 13.67, *p* < 0.001; Figure 8A2] and aggression [*F*_(3, 36)_ = 6.84, *p* < 0.001; Figure 8A3]. Specifically, LAB-I mice spent more time in non-aggressive social investigation, whereas LAB-S mice showed more aggressive behavior than the other three lines (all *p* < 0.05).

With a validated protocol of PPI measurement, 13 HAB, 23 NAB, and 32 LAB mice were tested in PPI test to evaluate animals' sensorimotor gating. Figure 8B illustrate the relative changes of startle responses at different prepulse (PP) intensities. There were no significant differences between the lines at 55 dB (*F* < 1; Figure 8B1) and 65 dB (*F* < 1; Figure 8B2), but at 75 dB PP intensity [*F*_(3, 62)_ = 5.27, *p* < 0.001; Figure 8B3]. There was also a significant line × stimulus interval interaction [*F*_(12, 248)_ = 3.27, *p* < 0.001] at 75 dB PP intensity. In comparison with NAB mice, both LAB-I and LAB-S mice displayed pronounced PPI at IPI of 10 ms (*p* < 0.01), whereas HAB mice showed less PPI with IPI of 25 ms (*p* < 0.01). In the pulse-alone trials (115 dB), the One-Way ANOVA revealed significant group differences [*F*_(3, 62)_ = 32.20, *p* < 0.001; Figure 8B1, inset], with LAB-I and LAB-S mice displaying higher, and HAB mice lower startle responses compared to NAB mice (all *p* < 0.001).

### LAB mice display elevated arousal and active coping styles

**Figure 9:**
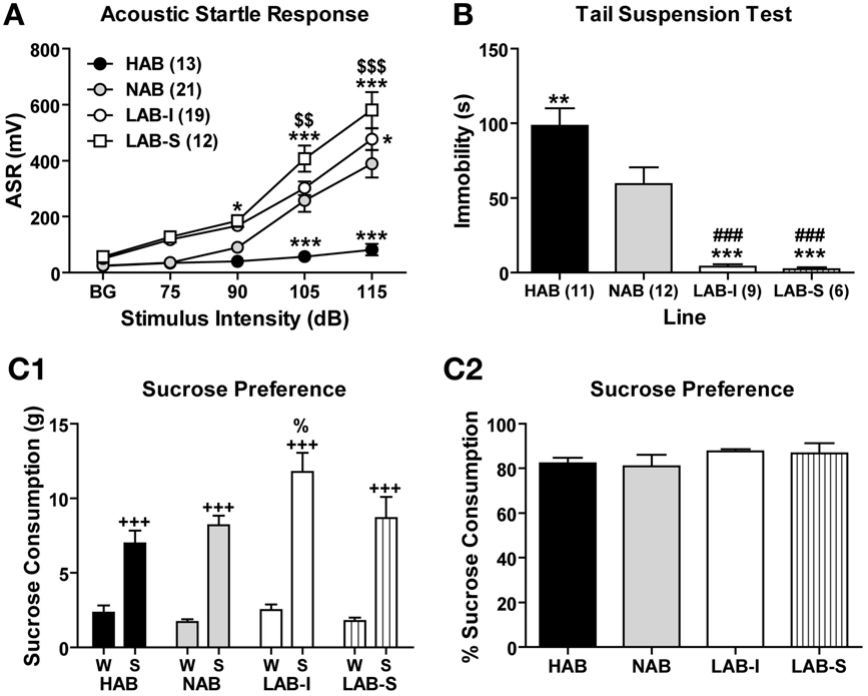
**LAB mice revealed hyperarousal and active coping styles, but did not show elevated sucrose preference. (A)** Acoustic startle responses (ASR) were elicited by acoustic stimuli. LAB-I and LAB-S mice showed higher, and HAB mice lower startle responses than NAB mice. **(B)** In the tail suspension test, HAB mice displayed more, but LAB-I and LAB-S mice less immobility than NAB mice. Mean ± SEM. ^***^p < 0.001; ^**^p < 0.01; ^*^p < 0.05 *vs*. NAB. ^###^*p* < 0.001 *vs*. HAB. ^$$$^*p* < 0.001; ^$$^*p* < 0.01 *vs*. LAB-I (ANOVA followed by *post-hoc* Newman–Keuls test). In the sucrose consumption test, **(C1)** animals of all four lines showed a preference for 2.5% sucrose. Moreover, LAB-I mice consumed more 2.5% sucrose than the other three lines (%, *p* < 0.05). **(C2)** In addition, percentage of sucrose consumption did not differ between all four lines. W, water; S, sucrose. ^+++^*p* < 0.001 compared with W (ANOVA followed by *post-hoc* Newman–Keuls test).

Given the *a priori* differences in ASR revealed during PPI measurements, we systematically assessed ASRs evoked by different startle intensities (Figure 9A). LAB-I and LAB-S showed stronger startle responses to the startle pulses of higher intensities than NAB and HAB mice, thus confirming our previous observations (Yen et al., 2012). The Two-Way ANOVA revealed significant differences between the four lines [*F*_(3, 61)_ = 19.92, *p* < 0.001] with a significant line × intensity interaction [*F*_(12, 244)_ = 12.50, *p* < 0.001]. *Post-hoc* analyses revealed that the ASR were higher in LAB-S mice than in HAB and NAB mice at 90, 105, and 115 dB (*p* < 0.05) and than in LAB-I mice at 105 and 115 dB (*p* < 0.01), as well as in LAB-I compared to NAB mice at 115 dB (*p* < 0.05). Moreover, HAB mice showed lower startle responses than NAB mice at 105 and 115 dB (*p* < 0.001).

In the TST, LAB mice displayed significantly less immobility time than HAB and NAB mice [*F*_(3, 34)_ = 20.76, *p* < 0.001; Figure 9B], with HAB mice showing the highest, NAB mice intermediate and LAB-I/LAB-S mice the lowest levels of immobility, similar to the findings by Krömer et al. (2005) and indicative of increased active coping.

### LAB mice do not reveal elevated preference for sucrose

In a two-bottle choice task, the Two-Way ANOVA revealed main effects of line [*F*_(3, 34)_ = 4.51, *p* < 0.01] and bottle [*F*_(1, 34)_ = 154.81, *p* < 0.001] as well as significant line × bottle interactions [*F*_(3, 34)_ = 3.75, *p* < 0.05]. *Post-hoc* comparisons showed that mice of all four lines showed a pronounced preference for the 2.5% sucrose solution relative to water (*p* < 0.001), but LAB-I mice consumed more sucrose solution than HAB, NAB, and LAB-S mice (*p* < 0.01; Figure 9C1). For sucrose preference, results showed that percentage of sucrose consumption did not differ among four lines (*F* < 1; Figure 9C2).

### LAB mice show impaired social recognition

**Figure 10:**
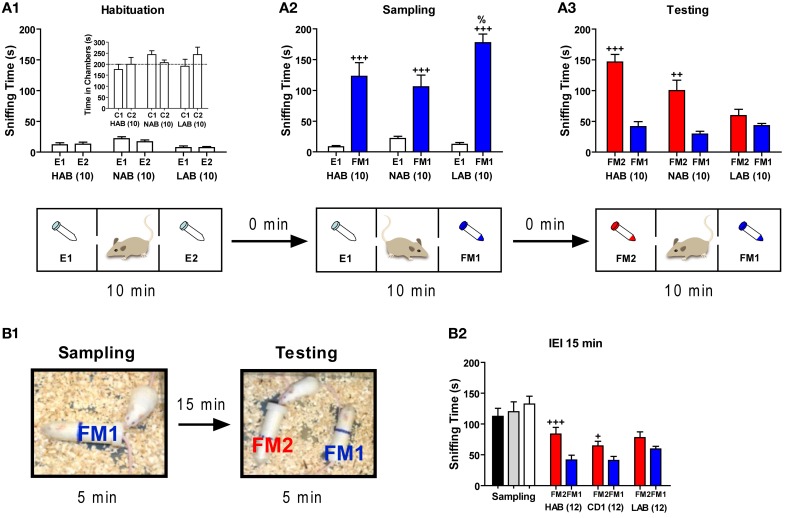
**LAB mice showed deficits in social cognition**. Mice of each line were assigned to three two social cognitive tests. Tests were performed with different batches of mice. **(A)** In a 3-chamber apparatus, mice were tested in three consecutive phases (each lasting 10 min): **(A1)** a habituation phase with empty 50 ml tubes in the left and the right chamber (E1, E2), **(A2)** a sampling phase, in which one empty tube was replaced by an identical tube containing a female (FM1), and **(A3)** a testing phase, during which the second empty tube was replaced by a tube containing another female (FM2). During the habituation phase, all three lines displayed similar investigation of the side chambers (C1, C2, inset) as well as sniffing time towards the two empty tubes, thus disclosing no side preference. All three lines spent more time in sniffing the female (FM1) exposed during the sampling phase compared to the empty tube (E1). LAB mice apparently lost interest in social investigation during the testing phase (as mirrored by the reduced total sniffing time) and failed to show a preference for the novel female (FM2). **(B1,B2)** In a social discrimination task, all three lines were introduced to an ovariectomized female (FM1) in an experimental chamber for 5 min, and after an interexposure interval (IEI) of 15 min, they were exposed to the familiar female (FM1) and a novel ovariectomized female (FM2) for another 5 min. Sniffing time did not differ between all three lines during the sampling phase. After 15 min, both HAB and NAB could discriminate between the FM1 and FM2, whereas LAB mice failed to do so. ^+++^*p* < 0.001; ^++^*p* < 0.01; ^+^*p* < 0.05 compared with E1 or FM1 (paired *t*-test). %*p* < 0.05 compared with HAB/NAB (FM1) (unpaired *t*-test).

Three groups of mice (10 HAB, 10 NAB, and 10 LAB) were subjected to the social preference test by using a 3-chamber apparatus. Again, LAB-I and LAB-S mice were merged as no performance difference was observed between two subgroups. During the 10 min of habituation phase, none of three lines showed side preference in the 3-chamber apparatus as reflected by the lack of differences between time spent in the left vs. the right chamber (*p* > 0.05; inserted in Figure 10A1). Moreover, no difference was found between time spent in sniffing two empty tubes (E1, E2) in the left and the right chambers (*p* > 0.05; Figure 10A1). In the next 10 min (i.e., the sampling phase), all three lines spent significantly more time in sniffing the tube containing an ovarectomized female mouse (social stimulus, FM1) than an empty tube [non-social stimulus, E1; *t*_(9)_ = −5.41, *p* < 0.001 for HAB; *t*_(9)_ = −4.83, *p* < 0.001 for NAB; *t*_(9)_ = −10.42, *p* < 0.001 for LAB; Figure 10A2]. Additional analyses revealed that LAB mice spent significantly more time in sniffing the social stimulus (FM1) than NAB and HAB mice [One-Way ANOVA: *F*_(2, 27)_ = 4.01, *p* < 0.05]. During the last 10 min (i.e., the testing phase), HAB and NAB mice spent significantly more time in sniffing the tube containing a novel ovarectomized female mouse (FM2) than that containing the familiar female mouse [FM1; *t*_(9)_ = −6.60, *p* < 0.001 for HAB; *t*_(9)_ = −4.10, *p* < 0.01 for NAB; Figure 10A3], confirming a preference for social novelty in HAB and NAB mice. In contrast, LAB mice failed to show such preference as indicated by the similar investigation duration of the novel and the familiar female mouse [*t*_(9)_ = −1.28, *p* = 0.23; Figure 10A3]. There was also a significant drop on the total time spent in investigating the social stimuli during testing compared to sampling in LAB mice, indicating a general loss of interest in social investigation.

In the social discrimination task, new batches of animals from all three lines were introduced to an ovarectomized female (FM1) for 5 min, and after different IEIs, they were exposed to the familiar female (FM1) and a novel ovarectomized female (FM2) for another 5 min (Figure 10B1). During the sampling phase, results showed that sniffing time did not differ significantly between the three lines (*F* < 1). With an IEI of 15 min (Figure 10B2), only HAB and NAB mice were able to discriminate between the familiar (FM1) and the novel (FM2) female [*t*_(11)_ = 3.93, *p* < 0.01 for HAB; *t*_(11)_ = 3.06, *p* < 0.05 for NAB], whereas LAB mice failed to discriminate two different social stimuli [*t*_(11)_ = −1.18, *p* > 0.05]. Interestingly, with an IEI of 2 h, only HAB mice could distinguish the novel female (FM2) from the familiar one [FM1; 94.3 ± 26.0 vs. 56.4 ± 26.2; *t*_(11)_ = −3.02, *p* < 0.05]. This ability vanished after an IEI of 4 h [82.6 ± 25.6 vs. 62.1 ± 30.3; *t*_(11)_ = 1.75, *p* > 0.05].

### LAB mice are impaired in spatial learning/reversal learning

**Figure 11:**
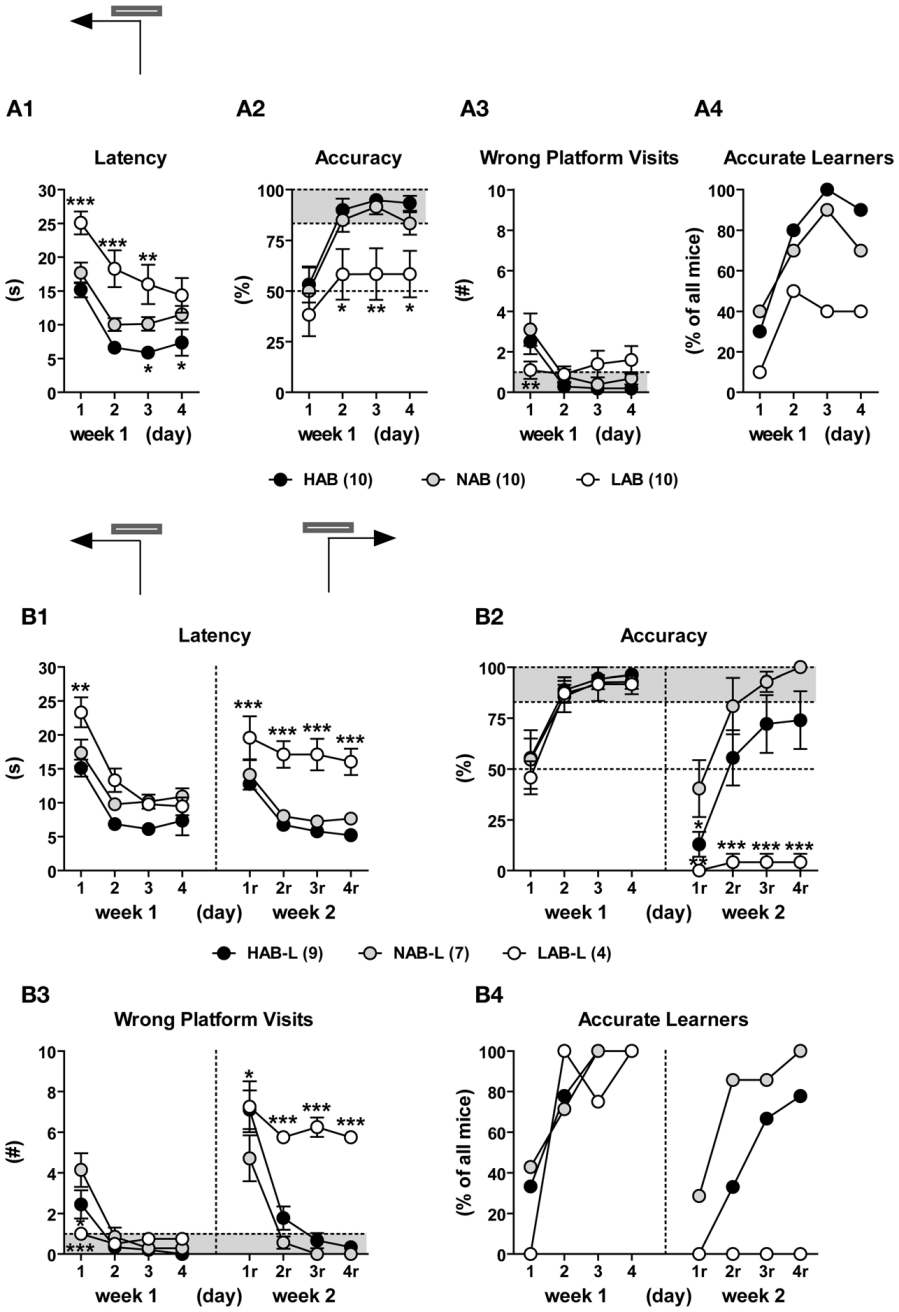
**LAB mice were impaired in spatial learning/reversal learning in the water cross-maze (WCM) test**. HAB, NAB, and LAB mice were started from the South arm of the WCM (which was resembled to a T-maze by blocking the entrance to the North arm) and trained to navigate to a hidden platform localized in the end of the West arm (d1–d4; week 1) or East arm (d1r–d4r; week 2), with 6 trials per day over the course of 4 consecutive days. Learning performance was described in terms of escape latency (i.e., time spent for reaching the platform), % accuracy (i.e., the percent accurate trials of 6 testing trial), number of wrong platform visits (i.e., number of entries of the outer third of the arm opposite to the goal arm), and number of accurate learners (i.e., animals that perform accurately in at least 5 or 6 trials per day). LAB-I and LAB-S were merged for analysis since both groups did not show differences in their general learning performance (data not shown). **(A)** Over the course of training during week 1, most HAB and NAB mice acquired that task, while LAB mice displayed impairment in acquisition. In the end of week 1, 9/10 HAB and 7/10 NAB but only 4/10 LAB had successfully acquired the task. **(B)** The accurate learners of week 1 (HAB-L, NAB-L, LAB-L) underwent reversal training, whereby the platform was located in the East arm (i.e., opposite to the original platform position). During relearning, 7 out of 9 HAB mice (78%) and 7 out of 7 NAB mice (100%) learned to correctly relocate the new platform position. In contrast, none of LAB mice displayed relearning. It is of note that LAB mice showed a remarkable preservation of the original platform position, which became evident from the high number of wrong platform visits (B3). Mean ± SEM. ^***^p < 0.001; ^**^p < 0.01; ^*^p < 0.05 *vs.* NAB (ANOVA followed by *post-hoc* Newman–Keuls test).

Ten HAB, 10 NAB, and 10 LAB mice were trained in a WCM test. LAB-I and LAB-S mice were merged as there was no performance difference between two subgroups. During the first week of training, all three lines showed a decrease in escape latencies [*F*_(3, 81)_ = 45.42, *p* < 0.001], implying learning of the task. However, significant differences were found between lines [*F*_(2, 27)_ = 10.65, *p* < 0.001] and further analysis revealed that escape latencies of HAB and NAB mice were significantly lower than those of LAB mice (*p* < 0.01; Figure 11A2). Statistical analysis also revealed a significant main effect of line in accuracy [*F*_(2, 27)_ = 5.41, *p* < 0.001; Figure 11A1]. Concerning the levels of accuracy on the last day of training (d4), 9 out of 10 HAB and 7 out of 10 NAB mice reached the accuracy criterion of ≥5 accurate out of 6 trials (83.3%), whereas only 4 out of 10 LAB mice reached the accuracy criterion (Figure 11A4). The number of wrong platform visits that is depicted in Figure 11A3 did not differ significantly among the three lines (*F* < 1).

During the second week, only the accurate learners of week 1 underwent reversal training with relocation of the platform to the opposite arm. On the first day of reversal learning, all animals showed memory perseverance, as reflected by the high number of visits to the original platform position and the resultant increase in escape latencies and low levels of accuracy. Ongoing training led to progressing relearning in HAB and NAB mice; however, there was virtually no reversal learning in LAB mice (Figure 11B). This phenomenon was reflected by significant main effects of line for all three learning parameters [escape latency: *F*_(2, 17)_ = 20.28, *p* < 0.001; accuracy: *F*_(2, 17)_ = 11.24, *p* < 0.001; wrong platform visit: *F*_(2, 17)_ = 24.94, *p* < 0.001; Figures 11B1–B3]. Further analyses revealed that LAB mice displayed impairment in relearning, as reflected by consistently high escape latencies, poor accuracy and a high number of wrong platform visits. Notably, on day 4 of reversal learning, all LAB mice still showed perseverance of the original platform position, as indicated by ≤1 accurate out of 6 trials and 5–6 wrong platform visits (Figures 11B2,B3).

### Haloperidol and lithium suppress hyperlocomotion in LAB mice

**Figure 12:**
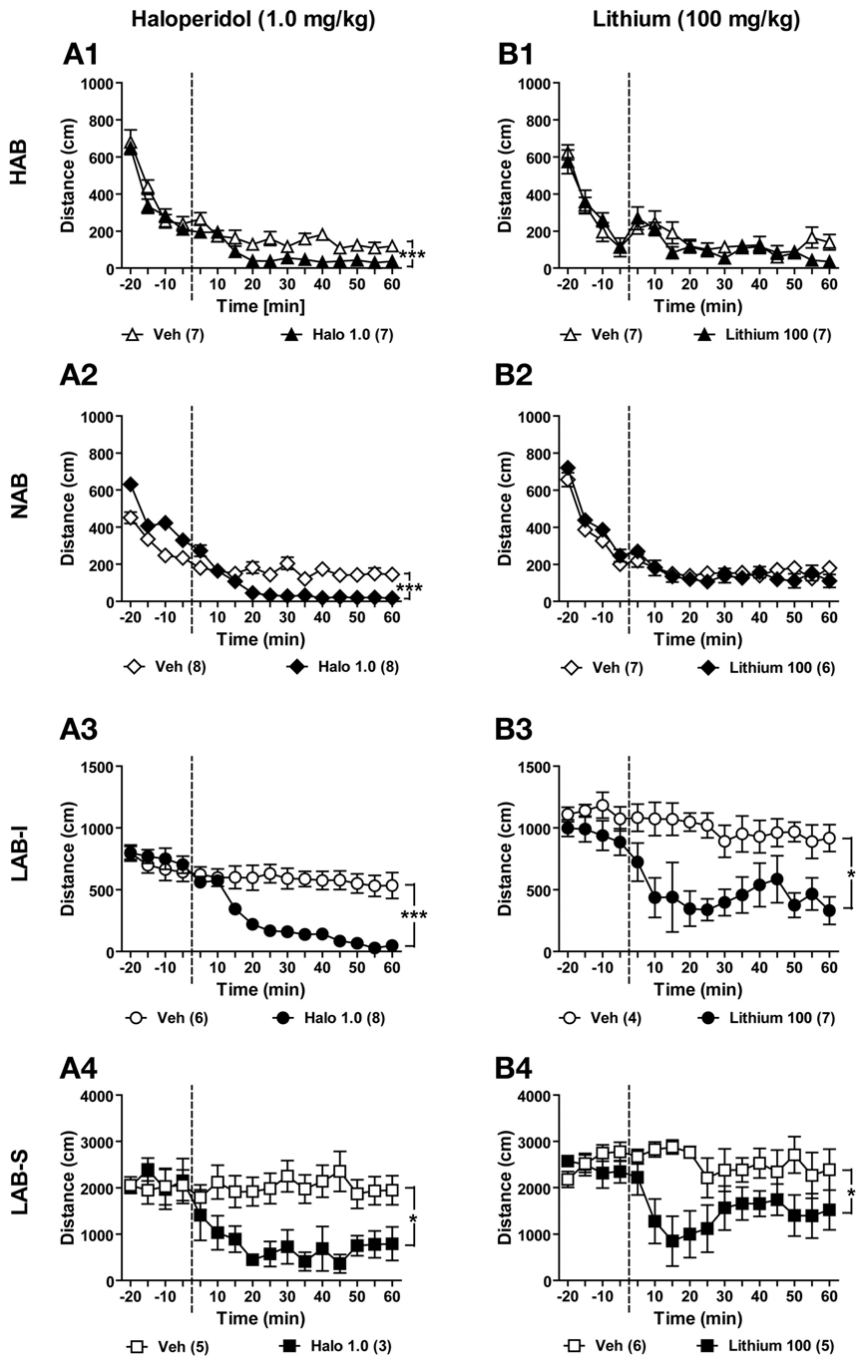
**Haloperidol and lithium suppressed locomotor activity in LAB mice**. Mice of all four lines were used for testifying the effects of haloperidol and lithium. **(A1–A4)** After 20 min of baseline exposure to the OF, HAB, NAB, LAB-I, and LAB-S mice were treated with either saline (Veh) or haloperidol (Halo) at 0 min (dashed lines). Treatment with haloperidol (1.0 mg/kg) significantly reduced locomotion in all mice. **(B1–B4)** New batches of mice were treated with either saline (Veh) or lithium (Lithium) at 0 min (dashed lines), and distance traveled was continuously measured for 60 min. Lithium (1.0 mg/kg) specifically decreased locomotion in LAB-I and LAB-S mice, but did not induce any change of locomotion in HAB and NAB mice. Mean ± SEM. ^***^p < 0.001; ^*^p < 0.05 compared with Veh (ANOVA followed by *post-hoc* Newman–Keuls test).

Haloperidol is an antipsychotic used to treat schizophrenia. To examine the effects of haloperidol on locomotion, two different batches of HAB, NAB, LAB-I, and LAB-S mice were tested in the OF test. Before drug administration (i.e., −20 to 0 min), no differences were observed between the two treatment groups per line, except for NAB mice [*F*_(1, 14)_ = 28.34, *p* < 0.001]. Fortunately, this difference did not impede the explanation of drug effects. Haloperidol (1.0 mg/kg) significantly suppressed locomotor activity in HAB [*F*_(1, 12)_ = 20.51, *p* < 0.001; Figure 12A1], NAB [*F*_(1, 14)_ = 33.77, *p* < 0.001; Figure 12A2], LAB-I [*F*_(1, 12)_ = 23.63, *p* < 0.001; Figure 12A3], and LAB-S [*F*_(1, 6)_ = 7.63, *p* < 0.05; Figure 12A4] mice.

Lithium is a classic mood stabilizer to prevent manic episode in bipolar disorder. In order to delineate the effects of lithium on locomotion, we administered lithium in mice of all four lines. During the 20-min OF exposure before lithium administration, no difference was found between lithium-treated and vehicle-treated animals (Figures 12B1–B4). Statistical analyses revealed that lithium (100 mg/kg) had no effects on locomotion in HAB [*F*_(1, 12)_ = 1.03, *p* > 0.05; Figure 12B1] and NAB mice (*F* < 1; Figure 12B2). In contrast, lithium treatment induced a significant reduction in locomotor activity in LAB-I [*F*_(1, 9)_ = 9.39, *p* < 0.05; Figure 12B3] and LAB-S [*F*_(1, 9)_ = 8.02, *p* < 0.05; Figure 12B4] mice.

### Amphetamine induced a paradoxical calming effect on locomotor activity in LAB-I and LAB-S mice

**Figure 13:**
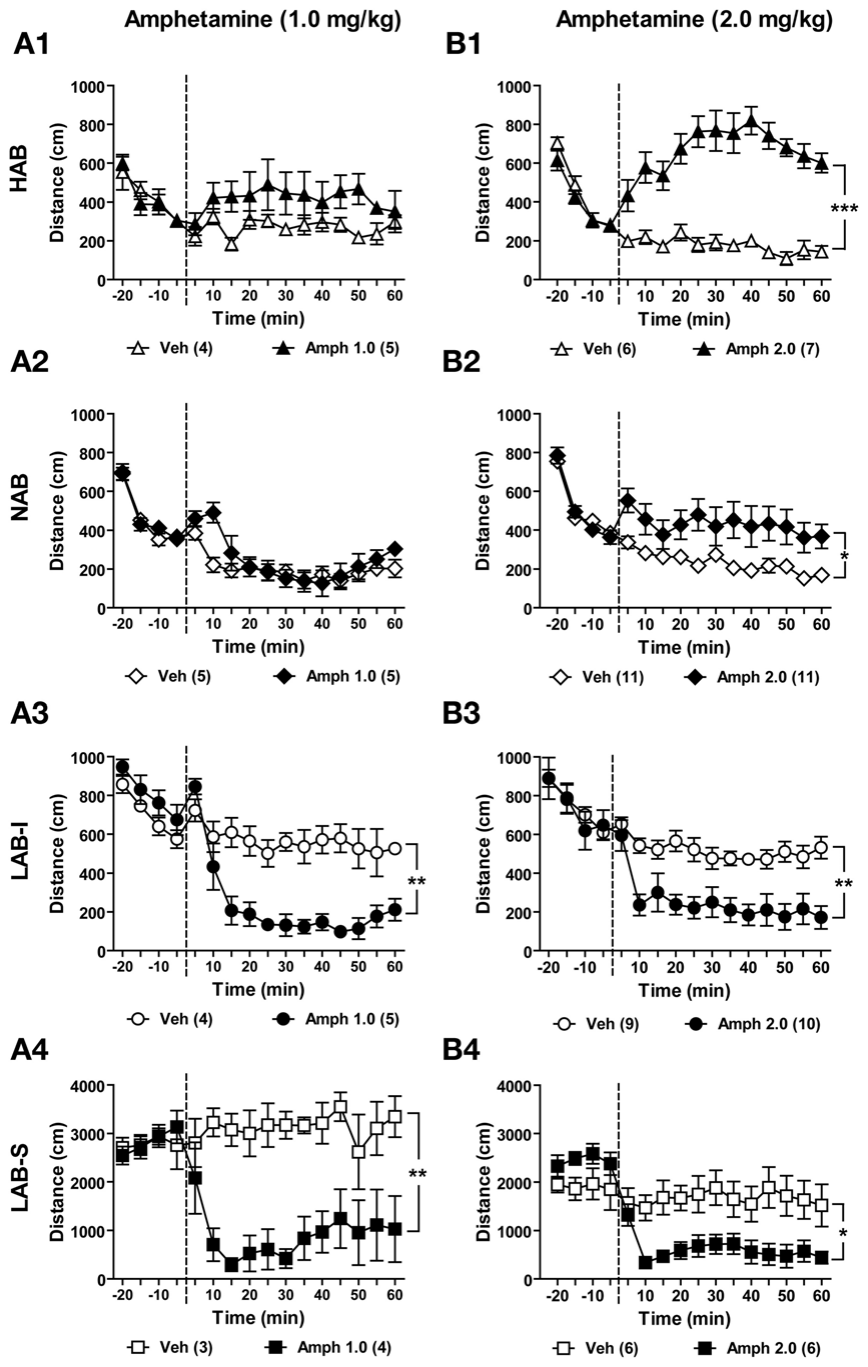
**Amphetamine induced a paradoxical calming effect on locomotor activity in LAB mice**. Mice of each line were randomly assigned to control (Veh) and amphetamine (Amph) groups. After 20 min of OF exposure, HAB, NAB, LAB-I, and LAB-S mice were treated with either saline or amphetamine at 0 min (dashed lines) and distance traveled was continuously measured for 60 min. Amphetamine of two different doses (1.0 and 2.0 mg/kg) were administered to separate groups of animals. **(A1–A4)** Treatment with amphetamine (1.0 mg/kg) did not affect the distance traveled in HAB and NAB mice. However, locomotor activity in LAB-I and LAB-S mice was attenuated by amphetamine at lower dose (1.0 mg/kg). **(B1–B4)** Treatment with amphetamine (2.0 mg/kg) induced strong increases in HAB, intermediate increases in NAB, and decreases in both LAB-I and LAB-S mice in distance traveled. Mean ± SEM. ^***^p < 0.001; ^**^p < 0.01; ^*^p < 0.05 compared with Veh (ANOVA followed by *post-hoc* Newman–Keuls test).

Amphetamine is commonly used for the treatment of ADHD, whereby it causes a paradoxical calming effect. Patients suffering from bipolar disorder, in contrast, show hypersensitivity to amphetamine. To investigate the effects of amphetamine at two different doses (1.0 and 2.0 mg/kg) on locomotion, different batches of HAB, NAB, LAB-I, and LAB-S mice were tested in the OF test and treated either with vehicle or with amphetamine (1.0 or 2.0 mg/kg). Mice of each line were assigned to control (Veh) and amphetamine (Amph) groups based on the locomotion data in the previous baseline test. Before amphetamine administration (from −20 to 0 min), no difference was observed between the two treatment groups per line (*p* > 0.05). Analyses of distance traveled showed that amphetamine at a dose of 1.0 mg/kg (i.p.) had no significant effects on distance traveled in HAB [*F*_(1, 7)_ = 2.18, *p* > 0.05; Figure 13A1] and NAB (*F* < 1; Figure 13A2) mice. However, decreased distance traveled was observed in LAB-I [*F*_(1, 7)_ = 21.15, *p* < 0.001; Figure 13A3] and LAB-S [*F*_(1, 5)_ = 17.94, *p* < 0.001; Figure 13A4] mice. The Two-Way repeated measures ANOVA revealed that administration of amphetamine (2.0 mg/kg, i.p.) resulted in increased distance traveled in HAB [*F*_(1, 11)_ = 44.83, *p* < 0.001; Figure 13B1] and NAB [*F*_(1, 20)_ = 6.75, *p* < 0.05; Figure 13B2] mice. Again, amphetamine treatment significantly decreased elevated levels of distance traveled in both LAB-I [*F*_(1, 17)_ = 14.34, *p* < 0.01; Figure 13B3] and LAB-S [*F*_(1, 10)_ = 8.39, *p* < 0.05; Figure 13B4] mice.

### Hyperarousal, active coping styles, and cognitive impairments in LAB mice cannot be rescued by acute amphetamine treatment

#### Effects of amphetamine on hyperarousal

**Figure 14:**
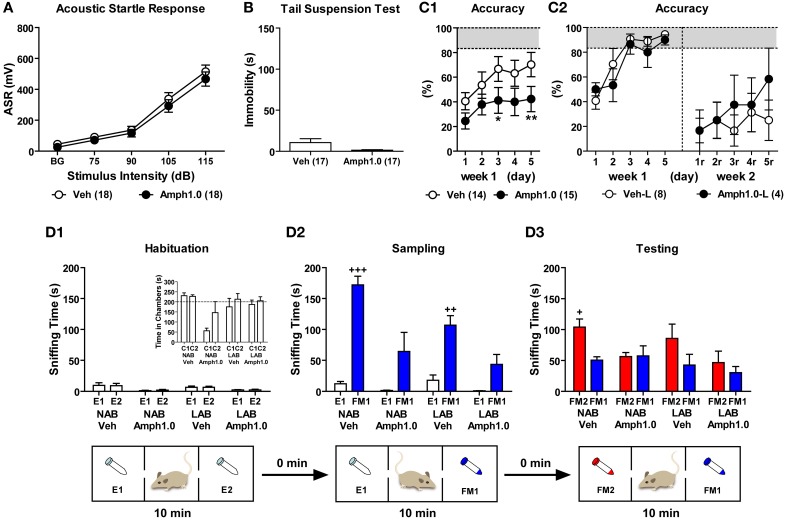
**Amphetamine failed to rescue the active coping styles and cognitive impairments in LAB mice**. To evaluate the effects of amphetamine on coping styles, LAB mice were randomly assigned to control (Veh) and amphetamine (Amph) groups and were tested in the startle response test and tail suspension test. LAB-I and LAB-S were merged for analysis since the two groups did not show difference in their sensation upon the acoustic stimuli and immobility (data not shown). Results revealed that treatment with amphetamine (1.0 mg/kg) did not influence **(A)** the startle responses and **(B)** immobility time in LAB mice. **(C)** To investigate the influence of amphetamine on spatial learning, LAB mice were trained in the WCM test with free learning protocol (cf. Figure 11). On each testing day 5 min before the first trial, animals were treated with either saline or amphetamine (1.0 mg/kg) and tested with 6 trials per day over the course of two weeks. **(C1)** During the first week of acquisition phase (d1–d5), the performance of learning acquisition was represented by accuracy and the treatment with amphetamine resulted in decreased levels of accuracy. **(C2)** Only 8/14 (57%) Veh-control and 4/15 (27%) Amph-treated LAB mice successfully acquired the test in the first week. These accurate learners (Veh-L, Amph1.0-L) were tested for the reversal training. During the relearning (d1r–d5r, right panels), both Veh-control and Amph-treated LAB groups failed to show relearning with no effect of amphetamine. Mean ± SEM. ^**^p < 0.01; ^*^p < 0.05 compared with Veh (ANOVA followed by *post-hoc* Newman–Keuls test). **(D)** To elucidate whether amphetamine is able to improve social recognition in mice, 9 NAB and 9 LAB mice were treated with either saline or amphetamine (1.0 mg/kg) immediately before introduction into the 3-chamber apparatus (cf. Figure 10A). **(D1)** During the habituation phase, there were no differences in sniffing two empty tubes irrespective of the lines and treatments, indicating no side preference of these animals. **(D2)** NAB and LAB mice treated with saline spent more time in sniffing the female (FM1) exposed compared to the empty tube (E1) during the sampling phase, whereas mice treated with amphetamine failed to show significant preferences to the social stimulus. **(D3)** During the testing phase, NAB-Veh mice showed a preference for the novel female (FM2), whereas either LAB-Veh or Amph-treated NAB and LAB mice failed to prefer exploration of the novel social stimulus. Mean ± SEM. ^+++^*p* < 0.001; ^++^*p* < 0.01; ^+^*p* < 0.05 compared with E1 or FM1 (paired *t*-test).

Both LAB-I and LAB-S mice displayed hyperarousal in the ASR test and comparable active coping styles as reflected by less immobility in the TST (cf. Figures 9A,B). To evaluate the effects of amphetamine on hyperarousal, we treated LAB mice with amphetamine 5 min before measurement of their startle responses (total duration of startle measurements: 34 min). Before amphetamine treatment, we compared the baseline startle responses to acoustic stimuli in LAB-I and LAB-S mice and did not observe significant differences. Therefore, we merged LAB-I and LAB-S mice to assess the amphetamine effects. Results revealed that treatment with amphetamine (1.0 mg/kg) did not influence the startle responses in these animals (*F* < 1; Figure 14A).

#### Effects of amphetamine on active coping styles

To investigate whether amphetamine has an influence on immobility in the TST, LAB mice were treated with amphetamine 20 min before the test. Statistical analysis revealed that amphetamine (1.0 mg/kg) induced a tendency to further decrease immobility in LAB mice [*t*_(32)_ = 1.86, *p* = 0.072; Figure 14B].

#### Effects of amphetamine on spatial learning

To elucidate whether amphetamine treatment could reverse the spatial learning deficits in LAB mice, we treated LAB mice with amphetamine before training in the WCM test. 30 LAB mice were assigned to control (Veh) and amphetamine (Amph) groups based on their locomotor activity in the OF. One animal died during the acquisition phase and was excluded from analyses. Five minutes before the first trial, animals were treated with either saline or amphetamine (1.0 mg/kg) and tested with 6 trials per day over the course of 5 consecutive days (d1–d5). Against our expectation, animals treated with amphetamine showed even further impairments in acquisition compared to Veh-control [Two-Way ANOVA: *F*_(1, 27)_ = 3.57, *p* = 0.070; Figure 14C1]. This became also evident by the number of accurate learners at the end of testing (d5), with 8 out of 14 vehicle-treated (57%) but only 4 out of 15 amphetamine-treated (27%) LAB mice, those reached the criterion of accurate learners. During the second week, only animals that successfully learned during the first week of training were tested in the reversal training. One Veh-control animal and one Amph-treated animal died during the second week. Similar to the acquisition phase, animals were treated with either saline or amphetamine (1.0 mg/kg) 5 min before the first trial and trained with 6 trials per day over the course of 5 consecutive days (d1r–d5r). Neither Veh-control (Veh-L) nor Amph-treated (Amph1.0-L) groups showed appropriate relearning till the end of training (i.e., d5r). Treatment with amphetamine failed to induce an improvement of relearning performance in terms of accuracy levels (*F* < 1; Figure 14C2), escape latency and wrong platform visits (data not shown).

#### Effects of amphetamine on social recognition

Given the impairments in the social recognition tests, we finally assessed the consequences of amphetamine on social recognition in NAB and LAB mice. NAB and LAB mice were randomly assigned to vehicle control (Veh) and amphetamine (Amph) groups. Mice were treated with saline or amphetamine (1.0 mg/kg) immediately before being introduced into the 3-chamber apparatus. During the habituation phase, there were no differences in sniffing two non-social empty tubes (E1, E2) in the left and the right chambers (*p* > 0.05; Figure 14D1 panel). In the sampling phase, however, Veh-treated NAB and LAB mice spent significantly more time in sniffing a female mouse (FM1) than an empty tube [E1; *t*_(4)_ = −11.23, *p* < 0.001 for NAB-Veh; *t*_(4)_ = −5.23, *p* < 0.01 for LAB-Veh]. In contrast, no similar preference of the social stimulus could be statistically secured after amphetamine treatment [*t*_(3)_ = −2.07, *p* = 0.13 for NAB-Amph; *t*_(3)_ = −2.75, *p* = 0.07 for LAB-Amph; Figure 14D2]. During the testing phase, NAB-Veh mice spent significantly more time sniffing a novel female mouse (FM2) than the familiar one (FM1) [*t*_(4)_ = 4.04, *p* < 0.05], but LAB-Veh and Amph-treated animals of both lines failed to display preference for social novelty [*t*_(4)_ = 1.32 for LAB-Veh; *t*_(3)_ = −0.12 for NAB-Amph; *t*_(3)_ = 0.63 for LAB-Amph, all *p* > 0.05; Figure 14D3].

## Discussion

**Figure 15:**
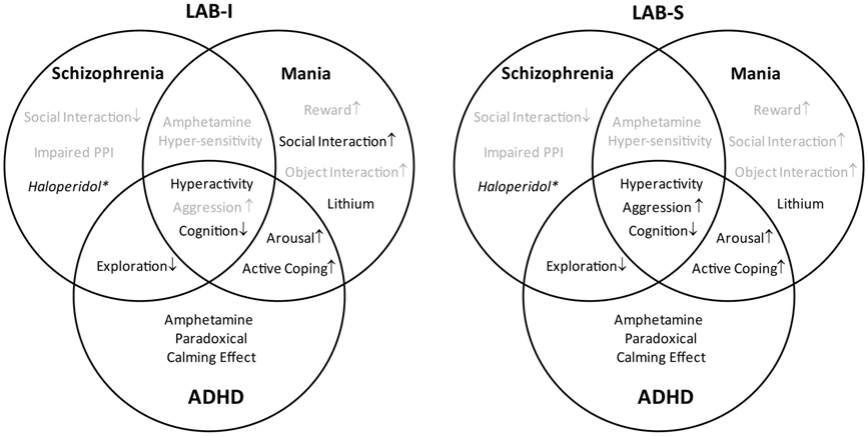
**Summary of the characteristics of LAB-I and LAB-S mice**. LAB-I and LAB-S mice are validated to possess some characteristics which may mimic the behavioral symptoms (face validity) and pharmacoresponsiveness (predictive validity) of three different psychiatric disorders (highlighted in black). As the diagram demonstrates, both LAB subgroups mainly fulfill the criteria of a valid animal model of ADHD. (^*^Haloperidol treatment attenuated locomotor activity in all mouse lines).

The present study demonstrates that mice selectively bred for low anxiety-related behavior (LAB) show robust hyperactivity without habituation as reflected by enduring increases in horizontal locomotion and mobility over the course of repeated OF exposures. With the phenotype of hyperactivity, LAB mice are proposed as an animal model of hyperactivity disorders, i.e., schizophrenia, mania, and ADHD. On the basis of human diagnostic criteria, we arranged the various endophenotypes in a Venn diagram and translated them into different behavioral tests. In order to validate the corresponding animal model, we systematically examined the mouse lines with respect to distinct and overlapping endophenotypes of these three diseases. The results of present study are summarized in Figure 15. Both LAB-I and LAB-S mice reveal hyperactivity and cognitive deficits, symptoms shared by the three diseases. LAB subgroups (especially LAB-S) show less exploration to the immediate environment, the overlapping symptom of schizophrenia and ADHD; however, these animals display no impairments in social interaction and PPI, implying the unlikelihood of LAB as an animal model of schizophrenia. Although LAB mice display hyperarousal and active coping styles, they fail to show the classic manic endophenotypes, such as increased social interaction, sucrose preference, and locomotor response to amphetamine. In fact, amphetamine even selectively reduced hyperactivity in LAB mice. Given the distinct behavioral profiles and pharmacoresponses, we conclude that LAB mice (in particular LAB-I) show many similarities to human ADHD and may, thus, serve as a novel animal model of this complex disorder.

In the EPM test, HAB and LAB mice displayed extremes in anxiety-related behavior, while NAB mice showed an intermediate level of anxiety-related behavior, confirming the previous findings (Krömer et al., 2005; Bunck et al., 2009). LAB mice seem to be novelty-seeking/risk taking and/or disturbed in behavioral inhibition, as reflected by a preference for open arm exploration. This finding is in line with recent observations about repetitive exploration at the edge in the cliff avoidance reaction (CAT) testing, which has been suggested as a measure of impulsivity (Yamashita et al., 2013). LAB mice traveled longer distances than HAB mice on the EPM (Figure 1B), suggesting differences in locomotion between two extreme lines. In order to investigate inter-individual differences in general exploratory behavior, mice were tested in the OF test. In agreement with the previous observation of increased home cage activity (Krömer et al., 2005), LAB mice were found to be more active than NAB and HAB mice, as could be seen by higher levels of horizontal locomotion and mobility in the OF test. Interestingly, two subgroups (LAB-S and LAB-I) can be distinguished in the LAB population. LAB-S mice displayed much higher levels of horizontal locomotion compared to HAB and NAB as well as LAB-I mice. Further, HAB and NAB showed habituation within and between daily tests, whereas both LAB subgroups failed to habituate to the testing environment as reflected by consistent levels of locomotion and mobility over the course of repeated exposures. The lack of habituation to the testing environment suggests that LAB mice might suffer dysfunctions of the hippocampus and/or mPFC, as both brain structures are involved in habituation processes (Godsil et al., 2005; Sarantis et al., 2012).

Analysis of LAB mouse individual locomotion separately by breeding pairs revealed that LAB-S mice were not originating from particular maternal/paternal combinations. Instead, the occurrence of LAB-S mice was equally distributed among these batches. This suggests that the bimodal phenotypes in LAB population cannot be ascribed to genetic drift restricted to a few breeding pairs nor to differences in maternal behavior. Instead, it might relate to heterozygosity in the general LAB population, which would result in homozygosity in 25% of the offspring. Yet, our data do not support such a scenario, since (i) LAB-S occurred with an incidence rate of 38% (far more than the predicted 25%), and (ii) LAB-I may turn into LAB-S upon repeated testing (which would argue against a genetic preponderance in defining the LAB-S phenotype) (Yen, unpublished observations).

In a recent study of genetic mapping for behavioral traits, general locomotor activity has been shown to be highly heritable across three different arena-based tests (Logan et al., 2013). To further investigate the heredity of hyperactivity, we cross-bred HAB and LAB mice. F1 offspring displayed relatively low levels of exploratory behaviors in the OF test similar to HAB mice irrespective of the maternal genotype. This provides evidence for the preponderance of genetic vs. epigenetic (i.e., maternal behavior) factors in determining hyperactivity as shown by LAB-I mice. Similar findings have been obtained in terms of anxiety-related behavior in HAB and LAB rats (Wigger et al., 2001). Cross-breeding of HAB and LAB rats resulted in F1 and F2 offspring displaying an intermediate level of anxiety in the EPM. In the present study, there were no significant differences in F1 hybrids originating from HAB × LAB vs. LAB × HAB breeding pairs. Together with the absence of hyperactivity, these findings indicate that the preponderance of the hyperactivity trait requires homozygosity, probably in recessive alleles, with no evidence for an additional role of genetic imprinting or maternal influences, at least on the hybrid background. The lack of maternal effects on locomotion is particular surprising, given the difficulties of LAB dams in maternal care (Kessler et al., 2010). Further experiments using prenatal (embryo transfer) and postnatal (cross-fostering) approaches with mice on a homozygous LAB background are necessary to further elucidate the roles of prenatal environment and mother-infant interaction in shaping hyperactivity in LAB mice.

In addition to hyperactivity, increased social aggression and cognitive dysfunction are also shared by schizophrenia, mania, and ADHD (Nevels et al., 2010; Gamo and Arnsten, 2011). During the social interaction test, elevated levels of social aggression were observed in LAB-S mice. Likewise, LAB rats displayed higher intermale aggression compared to normal Wistar and HAB rats during a resident-intruder test (Veenema et al., 2007). These findings support the notion that innate anxiety and aggression in rodents are inversely related and may be influenced by the same gene (Nyberg et al., 2003). Moreover, LAB mice were heavily impaired in a variety of cognitive tests, including WCM and social recognition tests. In the WCM experiment, general processes of spatial learning and memory turned out to be impaired in LAB mice. During the first week of training, 60% of LAB mice failed to acquire the task. Since the free choice learning protocol leaves it to the animals whether they acquire the task on the basis of response-based or place-based strategies (Kleinknecht et al., 2012), LAB mice appear to be generally impaired in spatial navigation. The same holds true for reversal learning, whereby only accurate learners of week one were used. This time, the platform was localized in the opposite arm, and the animals have to navigate to it by inhibiting the response pattern established in the week before. Analysis of perseveration errors revealed that LAB mice consistently visited the former platform position even at the last day of training, thus displaying severe deficits in cognitive flexibility. These findings are in accordance with results of a previous study demonstrating that LAB rats are impaired in declarative memory performance by showing more wrong choices than HAB rats (Ohl et al., 2002). Since C57BL/6N mice with bilateral lesions of hippocampus were able to suppress exploration of the former platform position (Kleinknecht et al., 2012), the inability of LAB mice to inhibit inappropriate responses points to additional deficits in other brain structures, such as the prefrontal cortex (Cubillo et al., 2010). Intriguingly, similar perseveration problems were reported in a study in pigs diverging for their escape attempts in the Backtest (Bolhuis et al., 2004). Briefly, a pig was classified as high-resisting (HR) or low-resisting (LR) if it performed more or less than four escape attempts respectively in two tests. In a spatial discrimination (T-maze) task, HR pigs were less successful in reversal learning then LR pigs, which is consistent with poor behavioral flexibility in LAB animals. In addition to the impaired spatial learning and memory, LAB mice also displayed social recognition deficits by reflecting reduced social discrimination abilities and social memory. Compared to HAB and NAB mice, LAB mice failed to show social discrimination abilities and displayed inferior social memory. In the social preference test, LAB mice may lose interest in social investigation during the testing phase (as mirrored by the reduced total sniffing time) and/or fail to discriminate between the two females. Similarly, in the social discrimination test, they may be unable to maintain the olfactory information and then to discriminate two females properly.

In contrast to elevated locomotion and rearing, LAB mice showed less hole exploration in the HB test, which indicates their reduced exploration to the immediate environment. Interestingly, there was an inverse relationship between horizontal locomotion and the accuracy of hole exploration. These findings go beyond simple hyperactivity and provide further consistency with reduced and superficial exploration to the details of environment in patients with schizophrenia, but not mania (Perry et al., 2009). Patients with schizophrenia exhibit some distinct symptoms from ADHD and mania, including reduced social interaction and impaired sensorimotor gating, usually measured by PPI (Grillon et al., 1992; Pearlson, 2000). In the social interaction and social preference tests, LAB mice, like NAB and HAB mice, displayed intact social preference to the social stimulus, reflecting by elevated time in sniffing female mice. The evidence speak against that LAB mice have altered social interaction. Here, we reported that the PPI in LAB mice was mostly similar to that in HAB and NAB when the level was 5 or 15 dB above background. However, LAB mice even showed more pronounced PPI compared to NAB controls at a higher prepulse levels (25 dB above background), This phenotype likely relates to the hyperarousal shown by LAB mice which might result in a startle response to the prepulse alone. Taken together, the deficits in social interaction and sensorimotor gating, and the reduced rather than increased exploration of their immediate environment shown by LAB mice, speak against an animal model of schizophrenia or mania. This conclusion is further supported by the lack of increased sucrose preference, given that hedonia, such as increased reward seeking, provides a cardinal symptom of mania, but not schizophrenia and ADHD. Moreover, patients with mania exhibit increased object interaction when exploring a new environment (Perry et al., 2009). Again, this was not the case during the habituation phase of the social preference test, when LAB mice did not show increased interaction with the two empty tubes. Thus, while LAB mice display some overlapping features of mania and ADHD, they fail to reveal the classic manic phenotypes.

Several studies have shown that selective breeding for one particular behavioral trait often results in alteration of a whole set of different behavioral endophenotypes related to anxiety, depression, locomotor activity, social behavior and cognition [for a review see Pawlak et al. (2008)]. In case of our selective breeding approach, we captured extremes in emotional and cognitive reactivity at the same time. That is, HAB mice with a genetic predisposition to hyperanxiety exhibit passive (or reactive) stress coping (Krömer et al., 2005) and pronounced conditioned fear (Yen et al., 2012), while less anxious animals (LAB) mice adopted active (or proactive) coping styles and appeared to be hyperactive novelty seekers. These findings support our initial hypothesis of the co-segregation of hyperactivity/novelty-seeking behavior under selective breeding of low trait anxiety. In line with this reasoning, other endophenotypes such as elevated levels of social interaction and social aggression as well as decreased immobility in the TST were observed in the LAB subgroups. The aforementioned results indicate that the individual trait anxiety level is associated with distinct abilities to cope with environmental challenges. Besides stress coping styles, HAB and LAB mice also behaved quite differently in a variety of cognitive tests. Our previous studies show that HAB mice displayed a pronounced cued-conditioned fear, whereas LAB mice were impaired in fear acquisition by showing lower freezing levels (Yen et al., 2012). However, hyperactivity described in LAB mice can confound the interpretation of decreased levels of freezing behavior in fear conditioning. Converging the aforementioned evidence in different species, we suppose that the dichotomy of cognitive processing may also be interpreted by co-segregation of different behavioral features. Remarkably, this co-segregation was not only found in HAB/LAB mice but also in HAB/LAB rats. By the same bidirectional selective breeding approach, HAB and LAB rats also exhibit binominal behavioral phenotypes regarding to coping styles, emotionality, locomotion and cognition. Likewise, LAB rats displayed elevated startle responses (Yilmazer-Hanke et al., 2004), higher intermale aggression (Veenema et al., 2007) and inferior cognitive abilities (Ohl et al., 2002), compared to normal Wistar and HAB rats. Similarly, selective breeding for another trait (high- vs. low-novelty exploration or aggression vs. non-aggression) also co-segregates a number of features. That is, rats bred for high responses to novel environment are prone to be aggressive (Kerman et al., 2011), impulsive (Flagel et al., 2010), and abuse of self-administer drugs (Davis et al., 2008). Additionally, aggressive individuals show many similarities with the LAB mice, including active coping styles and impaired behavioral flexibility (Benus et al., 1991). These findings support the notion that the co-segregation observed in the present study did not emerge by serendipity, but reflect fundamental co-existence of different behavioral traits. The co-existence might be interpreted by the notion that the differential traits may not evolve in isolation, but rather as a package caused by pleiotropy, gene-linkage, or co-selection (Price and Langen, 1992).

To further determine the type of disease model, we examined animals' differential responses to medications representative for schizophrenia, mania, and/or ADHD. The locomotor activity of LAB mice was successfully attenuated by haloperidol, an antipsychotic drug for the treatment of schizophrenia. However, haloperidol caused a general decrease in locomotor activity, irrespective of the mouse line and, thus, cannot be used as a distinctive criterion of LAB mice. Lithium is a classic mood stabilizer commonly used to prevent manic episodes in bipolar disorder. Treatment with lithium selectively reduced hyperactivity in LAB, but not HAB and NAB, mice, indicating that LAB mice share some pathogenetic features with manic individuals. In humans, amphetamine has been shown to increase general activity in majority of normal adults and to precipitate manic episodes in bipolar disorders (Gould et al., 2001; Frey et al., 2006) and positive symptoms in schizophrenia (Irwin et al., 1987), while exerting a paradoxical “calming effect” in ADHD patients (Greenhill, 1992). In the present study, treatment with amphetamine increased locomotor activity in HAB/NAB mice, but exerted a calming effect on locomotion in both LAB-I and LAB-S mice. These findings strengthen the hypothesis of LAB mice as a new mouse model of ADHD rather than mania or schizophrenia. Interestingly, the response to amphetamine seemed to depend on the level of basal locomotor activity in the OF, with HAB mice being highly stimulated, NAB mice being intermediately stimulated, and LAB mice being depressed, which perfectly fits into the rate-dependent hypothesis (Davids et al., 2002; Solanto, 2002). This hypothesis predicts that stimulants increase or decrease activity, depending on baseline activity levels (Andersen, 2005). The exact nature of amphetamine action as well as the biological basis of the line differences remain to be elucidated in future studies.

Although our pharmacological experiments fulfill predictive validity of ADHD for LAB mice, the fact that we haven't studied attention deficits and increased impulsivity as core symptoms of ADHD represents a limitation of the present study. We are currently planning further studies by using the 5-choice serial reaction time task to evaluate animals' attention level.

In contrast to its striking calming effects on locomotion, amphetamine failed to affect hyperarousal and active coping styles (TST) as well as the deficits in spatial learning and social memory in LAB mice. These results indicate that these traits might be co-selected but regulated by different neurobiological mechanisms (e.g., increased locomotor activity *per se* does not account for decreased immobility in the TST). This is in line with results of clinical studies where psychostimulants exert paradoxical motor calming effects, but fail to reverse cognitive dysfunctions (Advokat, 2010) or improve the academic achievement (Advokat and Scheithauer, 2013) in ADHD patients. In rats, administration of amphetamine at low doses is able to restore attention in the PFC-lesioned rats. However, the same or escalating dosage of amphetamine causes impairments in memory (Chudasama et al., 2005). The trade-off of amphetamine effects on attention and memory may explain why the cognitive deficits in LAB mice cannot be rescued by amphetamine treatment. Accordingly, acute amphetamine treatment also impaired social memory in NAB mice. Clinical evidence points to sensitive phases in development (e.g., early childhood) when pharmacological treatment for ADHD is most efficient (Zito et al., 2000). Several studies have proposed that both the timing of treatment onset and treatment duration influence the therapeutic effects of the drugs (Andersen et al., 2002; Thanos et al., 2007; Britton, 2011). In our study, the timing of treatment may be too late to modify the cognitive deficits of adult subjects. Only very few studies in rodents have demonstrated the facilitating effects of psychostimulants on cognitive abilities (Shaywitz et al., 1978). Therefore, robust approaches have to be developed to assess the effects of psychostimulants and other clinically effective compounds in cognitive tasks relevant for ADHD. In this context, LAB mice may become particularly valuable.

In conclusion, on the basis of Venn diagrams (Figures 1, 2), we systematically examined the animals' behavioral features and responses to representative medications in order to determine which disorder is modeled by hyperactive LAB mice. The research strategy employed by the present study also provides an example for validation of candidate mouse models or for recognition of overlapping symptoms between disorders. Based on the behavioral and pharmacological profiles, LAB mice emerge as a new rodent model of ADHD (Figure 15). Further experiments are required to investigate the neuronal mechanisms underlying hyperactivity in LAB mice. Additionally, these animals would hold potential as tools for studying the pharmacogenetics of amphetamine treatment. Such studies may contribute to the development of more effective pharmacotherapies for psychiatric disorders linked to hyperactivity symptoms.

### Conflict of interest statement

The authors declare that the research was conducted in the absence of any commercial or financial relationships that could be construed as a potential conflict of interest.
